# Eigenmode-based approach reveals a decline in brain structure–function liberality across the human lifespan

**DOI:** 10.1038/s42003-023-05497-4

**Published:** 2023-11-07

**Authors:** Yaqian Yang, Shaoting Tang, Xin Wang, Yi Zhen, Yi Zheng, Hongwei Zheng, Longzhao Liu, Zhiming Zheng

**Affiliations:** 1 School of Mathematical Sciences, Beihang University, Beijing, China; 2 Key laboratory of Mathematics, Informatics and Behavioral Semantics (LMIB), Beihang University, Beijing, China; 3 Institute of Artificial Intelligence, Beihang University, Beijing, China; 4 State Key Lab of Software Development Environment (NLSDE), Beihang University, Beijing, China; 5 Zhongguancun Laboratory, Beijing, China; 6 Beijing Advanced Innovation Center for Future Blockchain and Privacy Computing, Beihang University, Beijing, China; 7 PengCheng Laboratory, Shenzhen, China; 8 Institute of Medical Artificial Intelligence, Binzhou Medical University, Yantai, China; 9 Beijing Academy of Blockchain and Edge Computing (BABEC), Beijing, China

**Keywords:** Network models, Cognitive ageing

## Abstract

While brain function is supported and constrained by the underlying structure, the connectome-based link estimated by current approaches is either relatively moderate or accompanied by high model complexity, with the essential principles underlying structure-function coupling remaining elusive. Here, by proposing a mapping method based on network eigendecomposition, we present a concise and strong correspondence between structure and function. We show that the explanation of functional connectivity can be significantly improved by incorporating interactions between different structural eigenmodes. We also demonstrate the pronounced advantage of the present mapping in capturing individual-specific information with simple implementation. Applying our methodology to the human lifespan, we find that functional diversity decreases with age, with functional interactions increasingly dominated by the leading functional mode. We also find that structure-function liberality weakens with age, which is driven by the decreases in functional components that are less constrained by anatomy, while the magnitude of structure-aligned components is preserved. Overall, our work enhances the understanding of structure-function coupling from a collective, connectome-oriented perspective and promotes a more refined identification of functional portions relevant to human aging, holding great potential for mechanistic insights into individual differences associated with cognition, development, and neurological disorders.

## Abstract (web summary)

An eigenmode-based structural and functional connectivity mapping approach applied to diffusion and functional MRI data reveals decreased structure-function liberality with age across the human lifespan.

## Introduction

The human structural connectome promotes communication among distributed cortical regions, giving rise to richly patterned neural synchrony that is thought to support a wide range of cognitive functions and behaviors^1,2^. Characterizing the relationship between brain structure and function is a fundamental question in neuroscience, which is instrumental for understanding how cognitive processes emerge from the underlying anatomical pathways and for advancing the treatments for neurological and psychiatric diseases^3^. With the development of network science and imaging techniques, brain structure–function relationships are increasingly investigated using macroscale structural connectivity (SC) and functional connectivity (FC) networks^4,5^, which characterize the physical pathways and temporal synchrony between brain regions, respectively. A number of studies^6–8^ have shown that there exists a significant correlation between these two measures, where SC appears to act as a skeleton that constrains FC.

Multiple models have been proposed to explore how the FC network is coupled with the SC network, ranging from the simplest one-to-one mapping^9^ using statistical correlations to more sophisticated biophysical models^10,11^ that derive functional connectivity from large-scale simulations of neural activity dynamics. Communication models^12,13^ fall between these two extremes, where functional connectivity is conceptualized as a weighted superposition of communication events over the structural network, with the forms of communication ranging from the shortest path routing (centralized) to signal diffusion (decentralized)^14^. This approach achieves higher accuracy than the direct correlation method and lower complexity than the biophysical models, and as a result, become increasingly common in SC-FC mapping studies. Besides, another appealing tool for SC-FC mapping is the eigenmode approach^15,16^. This approach exploits a simple linear model that represents the FC network as a weighted combination of structural eigenmodes but achieves a high prediction accuracy comparable to sophisticated nonlinear models. These eigenmodes summarize structural connectivity into frequency-specific spatial patterns, opening an avenue to explore structure–function relationship by decomposing functional signals into the eigenspectrum of the structural connectome^17–19^.

In addition to modeling advances, structure–function relationships have also been applied to investigate the effects of cognitive tasks^20,21^, lesions^22,23^, neurological disease^24,25^, development, and aging^26–28^. As one of the main goals of SC-FC mapping models is to capture the essential principle of how structure and function are related, a natural expectation is that the estimated structure–function relationships would exhibit behavioral relevance and could reflect the effects of manipulations and perturbations^29^. Indeed, some recent studies have revealed associations between SC-FC correlations and various cognitive traits. One such study shows that increased alignment between structure and function is related to better cognitive flexibility^20^. Other studies suggest that weaker SC-FC coupling is related to increasing awareness levels^25^ and better recovery after severe brain injury^23,30^. Moreover, the strength of structure–function coupling is demonstrated to be heritable and to vary with subjects’ sex and age^26–28,31^.

Although SC-FC mapping has been fruitfully investigated and widely applied, the current literature is subject to the relatively moderate correspondence between brain structure and function. SC rarely explains more than 50% of the variance in empirical FC^29^, which implies that, to a great extent, the mechanisms underlying the formation of functional connectivity remain elusive. Recently, a unified framework^15^ for eigenmode approaches reports that none of the tested models with structural inputs could outperform a reference mapping that simply returns the group-average FC matrix. This contrasts with the intuition that mappings using variations in structural connectivity could provide additional information not captured by the mean, implying that inter-individual variability may not be adequately captured. Another study^28^ comparing a large number of communication models shows that whole-brain FC is poorly predicted from the structure in individuals, irrespective of predictors. As strong alignment between predicted and empirical functional networks appears desirable to ensure the fidelity of the captured information, this modest explanatory power is unfavorable for the refined investigation of structure–function relationships and for further applications to individual differences associated with behavior and cognition.

Why this imperfect link between SC and FC? There exist two intriguing hypotheses. The first one is that the relationship between SC and FC may itself be decoupling to some extent. Several studies on regional structure–function relationships have shown that structure and function are tightly coupled in primary sensorimotor cortex but decoupled in polysensory association cortex^32,33^. This gradual divergence closely follows representational and cytoarchitectonic hierarchies, in parallel to a functional gradient^34^ that associated cortical organization with a spectrum of increasingly abstract cognitive functions, raising a possibility that the observed structure–function divergence may be a fundamental property of the brain organization. The alternative hypothesis is that SC and FC may be tightly coupled but current models leave out information requisite for precise prediction. Multiple studies have revealed the important roles of microstructural properties in functional interactions^35–37^, and the explanation of function is improved by incorporating information on gene co-expression^38^, raising the possibility that SC-FC correspondence could be enhanced by more nuanced models that encompass biological details. Indeed, recent studies^39,40^, using the machine learning approach and high-frequency eigenmodes, have enhanced structure–function coupling. Nevertheless, high accuracies of these approaches often come with high execution time and model complexity, and the essential principles of the FC organization are still unclear.

Therefore, whether, and if so, how to establish a simple and tight link between SC and FC remains an important unsolved issue in the investigation of structure–function relationships. Here, we attempt to shed light on this question with a mapping approach that interprets the essential pattern of functional interactions in the context of the structural eigenspectrum. Different from the previous SC-FC mappings that keep the interregional connectivity central, our approach concentrates on the inherent patterns of brain functional interactions, which not only effectively reduces the complexity of the mapping procedure but also may yield improved robustness against weak spurious connections induced by noise^41^. In this way, we aim to provide a concise and accurate quantification of brain structure–function relationships and show how SC-FC coupling changes over the human lifespan by applying the captured individual-specific information. Our analyses are organized as follows. We begin by presenting the proposed SC-FC mapping, analyzing both whole-brain and regional SC-FC coupling, and comparing the performance with the eigenmode approach and communication model. We then examine additional information not captured by the mean in terms of whether the subject-specific SC-FC mapping outperforms the mean FC mapping. Finally, we analyze how SC-FC relationships evolve across the human lifespan.

## Results

### SC-FC mapping through structural and functional modes

**Fig. 1:**
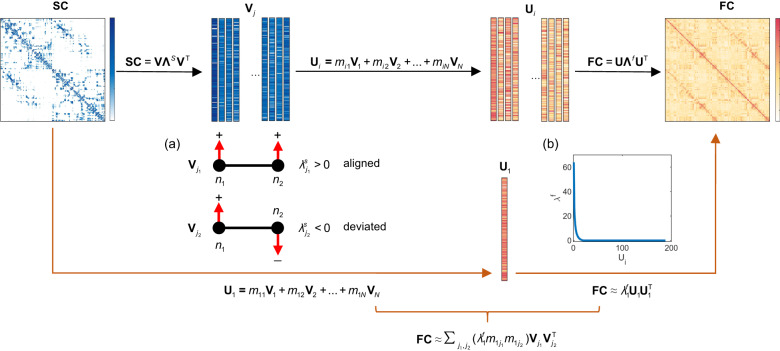
Method pipeline. Through the eigendecomposition of the structural network, we obtain a series of eigenmodes sorted in decreasing order of eigenvalues. The magnitudes of eigenvalues indicate the degree to which structural modes align with the underlying anatomy, with the large positive values corresponding to strict alignment and negative values corresponding to the deviation from SC. **a** illustrates a notion of the alignment of structural eigenmodes with respect to the underlying anatomical network. ${{{{{{{{\bf{V}}}}}}}}}_{{j}_{1}}$ indicates an anatomy-aligned structural eigenmode in which the values associated with nodes, represented by the directionality of the red arrows, align with that expected by the organization of anatomical connectivity. That is, highly connected regions possess similar values. In this toy example, connected brain nodes *n*_1_ and *n*_2_ contain values of the same signs. ${{{{{{{{\bf{V}}}}}}}}}_{{j}_{2}}$ indicates an anatomy-deviated structural eigenmode in which the values associated with nodes diverge from the underlying network, that is, highly connected regions possess values of different signs. In this example, connected nodes *n*_1_ and *n*_2_ possess values of different signs. These mutually orthogonal structural modes can be considered as a parsimonious basis for the empirical FC network which is also decomposed into its constituent eigenmodes accompanied by eigenvalues reflecting their contributions (black arrows). The distribution of functional eigenvalues of an observed FC network is displayed in (**b**). One can notice that the distribution of functional eigenvalues is far from uniform and that the FC network is dominated by a few functional modes with large eigenvalues. Accordingly, we approximate the observed FC by its most contributing functional mode (**U**_1_) which is fitted by a linear combination of structural eigenmodes to provide a concise mapping between the SC and FC networks (orange arrows).

As illustrated in Fig. 1, the eigendecomposition of the SC network provides a set of eigenvectors sorted in decreasing order of their eigenvalues, representing distinct inherent modes of the structural connectome. The alignment of these eigenmodes with respect to the anatomical connections is informed by their eigenvalues, characterizing to what extent the structural modes are organized aligned to or deviate from the underlying structural network^20^. Typically, the eigenvalue will be positive if the corresponding structural mode is strictly constrained by the underlying structural connectivity and negative if the structural mode is misaligned with anatomy (Fig. 1a; see Methods). We employ these mutually orthogonal structural eigenvectors as a parsimonious basis for the empirical FC network, which is also decomposed into its constituent eigenmodes. The functional eigenvalues reflect the contributions of corresponding functional modes to the FC formation, with larger values indicating greater contributions^42^. As shown in Fig. 1b, one can notice that this distribution is far from uniform and that the FC network is dominated by a few functional modes with large eigenvalues. Accordingly, we approximate the observed FC by its most contributing functional mode (i.e., the one with the largest functional eigenvalue) that is expressed as a linear weighted combination of structural eigenmodes to construct a concise mapping between SC and FC networks. The predictors are structural eigenmodes. The observation is the FC network that is approximated by the most contributing functional mode. Parameters can be easily evaluated in a closed-form manner (see Methods). Of note, while we focus on the leading functional mode here, further work could naturally incorporate more functional modes into the proposed SC-FC mapping and tune the balance of prediction accuracy and computational complexity according to the tasks. See Supplementary Note 1 for more details.

**Fig. 2:**
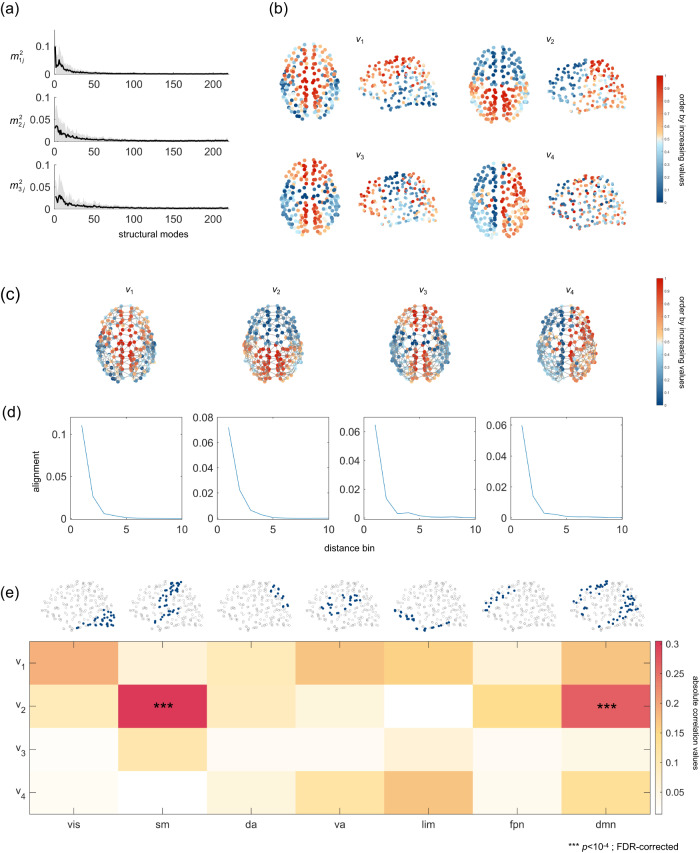
Analyses of highly contributing structural eigenmodes in LAU dataset. **a** The contribution of individual structural eigenmodes to the first three functional eigenmodes, which is quantified by the square of weights (${m}_{ij}^{2}$, $\mathop{\sum }\nolimits_{j = 1}^{N}{m}_{ij}^{2}=1$) obtained from the decomposition of individual functional modes in terms of the structural modes. The gray lines indicate the results for individual subjects and the black line displays the mean results across the subjects (*n* = 69 subjects). **b** The spatial distribution of the first four structural eigenmodes derived from the group-average structural connectome in LAU dataset. Brain nodes are colored from blue to red in ascending order of values. **c**, **d** Alignment of structural eigenmodes with respect to the structural connections of different distances. We divide structural connections into 10 distance bins in ascending order of their lengths (equal number of connections in each bin) and quantify the alignment of the first four structural modes with respect to the structural connections in each distance bin. **c** illustrates the spatial distributions of group-level (first four) structural eigenmodes with the top 5% short-distance connections, and (**d**) illustrates the alignment of group-level (first four) structural eigenmodes relative to connections of different distances bins. **e** The similarity between 7 canonical functional networks (RSNs) and group-level (first four) structural eigenmodes. RSNs include visual (vis), somatomotor (sm), dorsal attention (da), ventral attention (va), limbic (lim), frontoparietal (fpn), and default mode (dmn) networks. *** indicates FDR-corrected *p* < 10^−4^.

Given that the structural eigenspectrum is used to form a basis for FC prediction, we attempt to understand the structural eigenmodes that highly contribute to functional interaction patterns. To this end, we measure the contributions of individual structural eigenmodes to the first three functional eigenmodes, which are quantified by the square of weights obtained from the decomposition of functional modes into the structural modes^43^. As shown in Fig. 2a and Supplementary Fig. 1b, one can notice that brain functional modes are preferentially expressed by anatomy-aligned SC eigenmodes, i.e., those with large positive eigenvalues. We then display the physical distribution of the first four SC eigenmodes (Fig. 2b and Supplementary Fig. 1a and c). We find that the first four SC eigenmodes exhibit relatively low spatial frequencies. Specifically, we observe several main geometrical axes along which values associated with nodes vary (e.g., center-peripheral, anterior-posterior, anterior-middle-posterior, and left-right). We next calculate the alignment of SC eigenmodes with respect to structural edges within different distance bins (Methods) and find that the anatomy-alignment of the first four SC eigenmodes is predominately expressed by the organization of short-distance connections (Fig. 2c, d). Furthermore, to determine whether the spatial patterns of these structural modes are circumscribed by different functional systems, we assess their similarity to seven resting-state functional networks proposed by Yeo et al.^44^. Since an eigenmode is essentially identical under sign reversal, we utilize the absolute Pearson R. As a control, we perform a spin test^45,46^, in which we randomly rotate the nodes’ spatial locations while preserving brain spatial covariance structure (10,000 repetitions). We find that the 2nd structural eigenmode exhibited statistically significant similarity to somatomotor and default mode networks (Fig. 2e, FDR-corrected *p* < 10^−4^). We also associate structural eigenmodes with the first two functional gradient (G1_FC: unimodal-transmodal; G2_FC: visual-motor)^34^, the first three structural gradients (G1_SC: inferior-superior; G2_SC: anterior-posterior; G3_SC: medial-lateral)^47^, and the microstructural gradients (G1_hist: sensory/motor-transmodal/limbic; G1_mri: primary sensory-limbic)^37^. As illustrated in Supplementary Fig. 2, we find that the spatial patterns of structural eigenmodes closely resemble the distributions of structural gradients (e.g., high matching between *v*_1_ and G1_SC, *v*_2_ and G2_SC, *v*_3_ and G3_SC) and are somewhat associated with functional gradients and microstructural gradients (e.g., moderate similarity between *v*_1_ and G2_FC, *v*_1_ and G1_mri, *v*_2_ and G1_FC, etc.).

### Enhanced explanation of the FC network

**Fig. 3:**
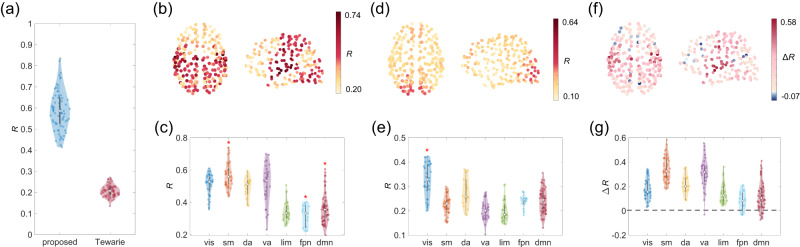
Whole-brain and regional performance of the proposed mapping. **a** The mapping performance of the proposed method vs. Tewarie et al.^16^ for individual subjects (*n* = 69 subjects) in LAU dataset. Metrics of performance are Pearson *R* between the estimated and empirical FC matrices, excluding the diagonal entries. In each violin plot, the box indicates the interquartile range and the empty circle indicates the median value. **b** The spatial pattern of regional SC-FC coupling estimated by the proposed method. **c** The distribution of *R* estimated by the proposed method over regions (*n* = 219 regions) aggregated by seven resting-state networks (RSNs); asterisks indicate FDR-corrected *p* < 0.01. **d** The spatial pattern of SC-FC coupling estimated by Tewarie et al. **e** The distribution of *R* estimated by Tewarie et al. over regions (*n* = 219 regions) aggregated by seven RSNs; asterisks indicate FDR-corrected *p* < 0.01. **f** The spatial pattern of regional differences between correlation *R* of the proposed mapping and Tewarie et al. (Δ*R* = *R*_proposed_ − *R*_Tewarie_). **g** The distribution of Δ*R* over regions aggregated by seven RSNs.

To assess the performance of the present method, we construct whole-brain SC-FC mapping for each subject from the LAU dataset and compare its performance with Tewarie et al.^16^ which possesses almost identical structural predictors and model complexity (see Methods). The Pearson correlation coefficient *R* between the upper-triangular part (excluding diagonal elements) of the predicted and empirical FC matrices is calculated to evaluate the mapping performance and the strength of structure–function coupling. As shown in Fig. 3a, we find that the proposed method yields significantly higher correlation than Tewarie et al. (proposed: *R* = 0.59 ± 0.09, Tewarie: *R* = 0.21 ± 0.03, paired *t* test *p* < 10^−10^). We also evaluate local SC-FC coupling by calculating the correlation between predicted and empirical FC profiles of brain regions (i.e., the corresponding rows of predicted and empirical FC matrices). As shown in Fig. 3b–e, we find that regional structure–function coupling varies considerably across the cortex for both approaches. Specifically, for the proposed SC-FC mapping, regions with high correlation are concentrated in the visual cortex, supertemporal cortex, and somatomotor (precentral and postcentral) cortices, whereas regions in the precuneus, cingulate, and prefrontal cortices exhibit relatively low prediction accuracy (Fig. 3b). To characterize these findings at the level of functional systems, we aggregate regional *R* by seven resting-state networks and compare the network-specific mean *R* to those generated by spatially constrained permutation (spin test; 10,000 permutations). We find that FC profiles of regions in the somatomotor network are better explained than the null distribution while FC profiles of regions in the frontoparietal and default mode networks are worse explained than explained by chance (FDR-corrected *p* < 0.01; Fig. 3c). Such system-specific effects also appear in regional SC-FC coupling estimated by Tewarie et al.^16^, with FC profiles of regions in the visual network significantly better explained than those of other regions (FDR-corrected *p* < 0.01; Fig. 3d, e). We further find the proposed method generally outperforms Tewarie et al. at the region-level, with the FC profiles of 76 ± 8% of regions being better explained (Fig. 3f, g). As our mapping contains both the linear combination of structural eigenmodes (which is also contained in Tewarie et al.) and the non-linear interactions of different structural eigenmodes, this observation highlights the strength of introducing non-diagonal interactions in eigenmode-based SC-FC mappings (see Methods for more details).

**Fig. 4:**
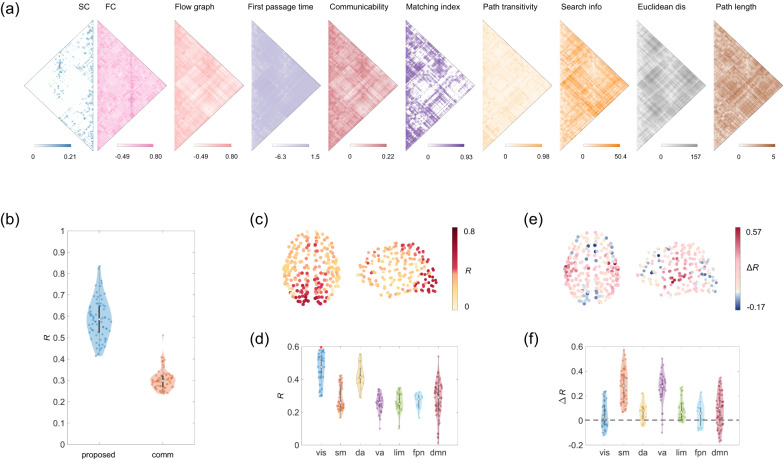
Comparison with the communication model. **a** A communication model that incorporates a large number of structurally informed predictors characterizing the geometric, topological, and dynamic relationships between regions. **b** The mapping performance of the proposed method vs. the communication model for all individual subjects (*n* = 69 subjects) in LAU dataset. In each violin plot, the box indicates the interquartile range and the empty circle indicates the median value. **c** The spatial pattern of regional SC-FC coupling estimated by the communication model. **d** The distribution of *R* estimated by the communication model over regions (*n* = 219 regions) aggregated by seven RSNs; asterisks indicate FDR-corrected *p* < 0.01. **e**, **f** Regional differences between correlation *R* of the proposed and communication methods (Δ*R* = *R*_proposed_ − *R*_comm_).

Next, we compare the proposed mapping with a communication model^14^ that incorporates a large number of predictors characterizing the geometric, topological, and dynamic relationships between regions (Fig. 4a; see Methods). As shown in Fig. 4b, we find that the correlation of the proposed method is significantly higher than that of the communication model (communication: *R* = 0.30 ± 0.04, paired *t* test *p* < 10^−10^). We also observe regional heterogeneity in structure–function coupling estimated by the communication model, with regions in the visual network exhibiting higher *R* values than regions in other functional systems (10,000 spatially constrained permutations, FDR-corrected *p* < 0.01; Fig. 4c, d). Furthermore, we show that the present method yields higher structure–function correspondence than the communication model across a wide range of cortex, with FC profiles of 67 ± 10% of brain regions being better explained by the proposed mapping (Fig. 4e, f). Collectively, these results indicate the validity of the proposed method, which is also verified in an independently collected dataset (NKI; Supplementary Figs. 3, 4).

### The advantage of capturing individual-specific information

**Fig. 5:**
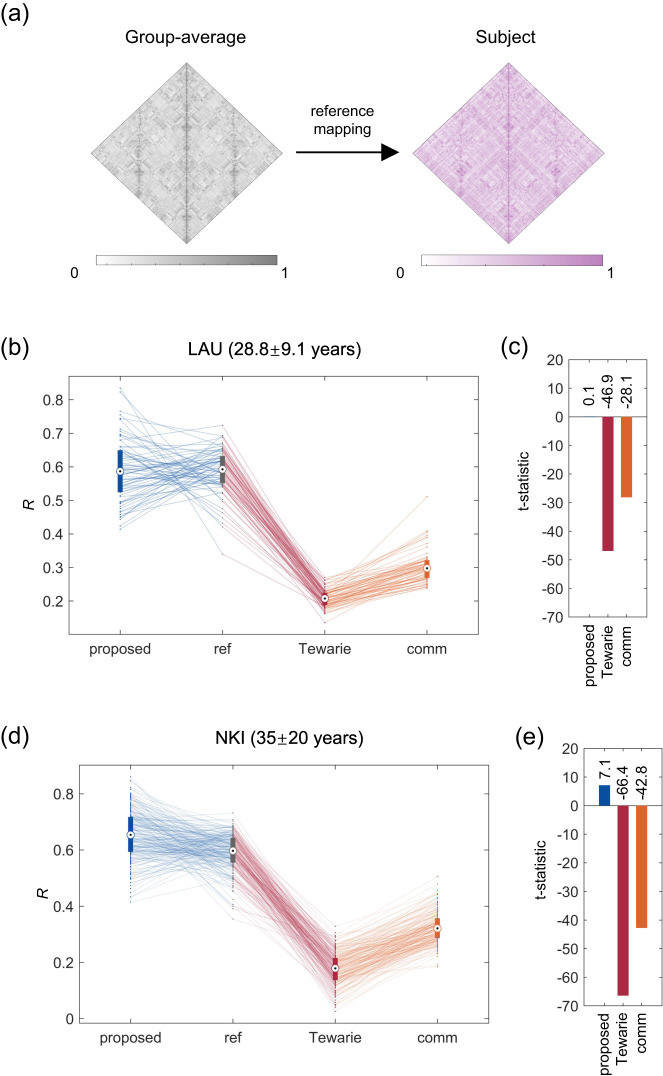
Individual-specific mapping between structural and functional networks. We introduce a reference mapping that always returns the group-average FC matrix to examine whether individual mappings utilizing structural information could capture additional information not explained by the mean. **a** illustrates a reference mapping that returns the group-average FC network. Specifically, the group-average functional connectivity matrix is constructed by averaging all individuals' FC matrices and the performance of the reference mapping is quantified by Pearson *R* between the group-average and individuals' empirical matrices (excluding diagonal elements). We then utilize this reference mapping performance to form a baseline, and compare the performance of individual mappings possessing structural inputs (i.e., the proposed method, Tewarie et al., and the communication model) against it. The performance of these individual mappings is evaluated by Pearson *R* between the estimated and empirical matrices (excluding diagonal elements), and the comparison is conducted via a paired *t* test. **b**, **c** illustrate the comparison results for an age-homogenous dataset (LAU, *n* = 69 subjects). **b** shows the mapping performance for individual subjects using different methods (the proposed method, the reference mapping, Tewarie et al., and the communication model). The boxplot shows the medians (circles), interquartile ranges (boxes), and min to max range (whiskers). Lines between different boxplots link the mapping performance of identical subjects across different methods. **c** shows the results of paired *t* test comparison, with the *y*-axis indicating the resultant *t* statistics. **d**, **e** illustrate the comparison results for a lifespan dataset (NKI, *n* = 196 subjects).

In this section, we seek to assess whether the proposed mapping is able to characterize inter-individual variation in functional connectivity. To this end, we introduce a reference mapping that simply returns the group-average FC matrix (Fig. 5a). This reference mapping neither utilizes structural information nor preserves inter-individual variation, thereby providing a benchmark against which the relative performance of individual-specific structure–function mapping could be measured.

We employ two independent datasets to perform the analyses. The first one is the LAU dataset, which consists of a homogeneous population of roughly the same age range (28.8 ± 9.1 years). The second one is the NKI dataset, which comprises a relatively heterogeneous population across the human lifespan (35 ± 20 years). For each dataset, we construct the group-average functional connectivity matrix by averaging all individuals’ FC matrices and quantify the performance of the reference mapping by Pearson *R* between the group-average and individuals’ empirical matrices (excluding diagonal elements). We then consider the performance of this reference mapping as a baseline and compare the performance of subject-specific mappings that make use of structural information (i.e., the proposed method, Tewarie et al., and the communication model) against it. The performance of these mappings with structural inputs is evaluated by Pearson *R* between the estimated and empirical matrices (excluding diagonal elements), and the comparison is conducted via a paired t-test. Fig. 5b–c and d–e illustrate the results on an age-homogenous dataset (LAU) and a lifespan dataset (NKI), respectively. Interestingly, we find that not all SC-FC mappings that utilize subject-specific structural information could outperform the mean mapping. Instead, in a homogeneous population, the mean mapping appears to serve as a glass ceiling, with the correlation *R* of both Tewarie et al. and the communication model significantly lower than that of the reference mapping (reference: *R* = 0.58 ± 0.06; paired *t* test *p* < 10^−10^; Fig. 5b, c). However, the proposed SC-FC mapping still performs well, achieving prediction accuracies comparable to the mean mapping (paired *t* test *p* = 0.90). Furthermore, in a heterogeneous population, the prediction accuracy of the proposed mapping is higher than that of the reference mapping whereas Tewarie et al. and communication methods still fail to outperform the reference mapping (paired t-test *P* < 10^−10^; Fig. 5d, e), indicating the advantage of the proposed method to capture additional subject-specific information not explained by the mean.

**Fig. 6:**
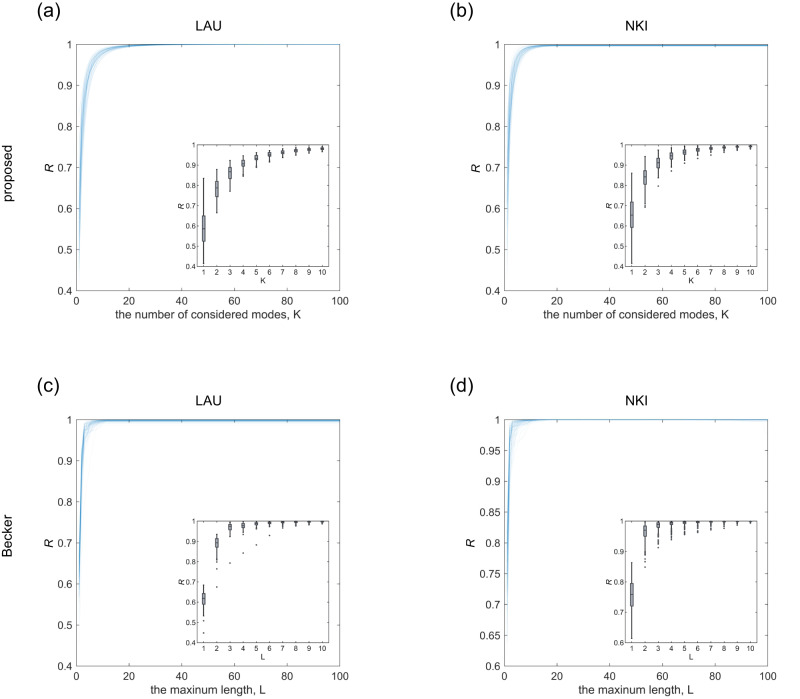
Comparison between the proposed mapping and Becker et al. **a** The evolution of mapping performance for individual subjects (*n* = 69 subjects) in LAU dataset using our method with different numbers of functional eigenmodes under consideration (from *K* = 1 to *K* = 100). Metrics of performance are Pearson *R* between the estimated and empirical FC matrices, excluding the diagonal entries. In the inset, we plot the boxplots of the proposed mapping performance when we vary the number of considered functional modes from *K* = 1 to *K* = 10. For each boxplot, the box indicates the interquartile range (IQR), the horizontal line indicates the median value, and the whiskers cover the upper and lower bound of 1.5 × *IQR* (25th and 75th percentiles). Outliers are beyond the whiskers, indicated by dots. **b** The evolution of the correlation *R* for individual subjects (*n* = 196 subjects) in NKI dataset using our method with different numbers of functional eigenmodes under consideration (from *K* = 1 to *K* = 100). **c** The evolution of the correlation *R* for individual subjects in LAU dataset using Becker et al. with the maximum length of the walks varying from *L* = 1 to *L* = 100. **d** The evolution of the correlation *R* for individual subjects in NKI dataset using Becker et al. with varying L (from *L* = 1 to *L* = 100).

We further extend the proposed mapping with the first K functional modes under consideration (see Supplementary Note 1) and find that incorporating more functional modes consistently yields improved explanatory power (Supplementary Fig. 5). We compare this extended mapping against the work from Becker et al.^48^ based on the maximum length L of structural walks (Fig. 6; Methods). We find that both methods provide high correlations (e.g., proposed: *R* = 0.97 ± 0.01 for *K* = 8; Becker et al.: *R* = 0.99 ± 0.00 for *L* = 8; LAU), with the differences that our approach has lower model complexity and better interpretability compared to Becker et al. (see Methods). Besides the above in-sample evaluation, we also introduce the HCP dataset^49^ which contains two sessions of functional magnetic resonance imaging (fMRI) data to assess the out-of-sample performance; the results are largely unchanged (Supplementary Fig. 6; Methods). We additionally compare the proposed approach with the Riemannian approach^50^ and find that both approaches achieve competitive performance as the values of K or walk length increase (e.g., the proposed: *R* = 0.76 ± 0.06 for *K* = 10; Riemannian: *R* = 0.76 ± 0.06 for walk length = 10; Supplementary Fig. 7).

### Weakened structure–function liberality across the human lifespan

In this section, we apply the proposed mapping approach to provide insights into how structure–function relationships evolve with age using the NKI dataset that comprises 196 healthy participants aged from 4 years to 85 years.

**Fig. 7:**
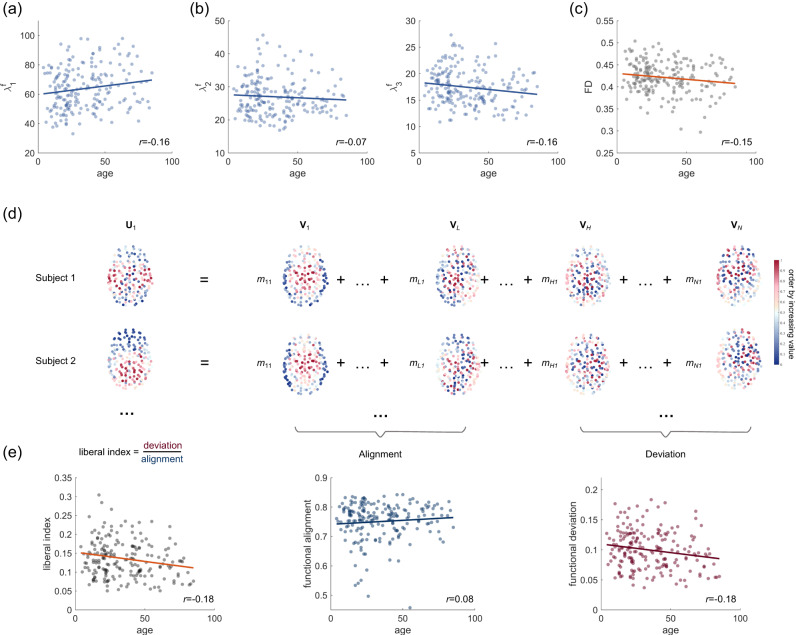
Age-related variations in SC-FC relationships. **a** The contribution of the first functional mode to the FC network increases with age. **b** Age-related variations in contributions of the 2nd or 3rd functional modes. **c** Decreases in functional diversity across the lifespan. **d** The functional interaction pattern for each subject is decomposed into structure-aligned and structure-deviated components in terms of the SC eigenmodes. The ratio between the norms of these two components is used to measure structure–function liberality. **e** Reduced structure–function liberality across the lifespan, which is predominantly driven by the weakened functional deviation.

As the proposed SC-FC mapping is constructed via highly contributing functional modes, we first assess whether and how their contributions to the FC network vary with age. To this end, we calculate the eigenvalues of the first three functional modes for each subject and correlate the values with their ages. Here, we are focusing on the first three functional modes since they almost explain the variance in empirical FC networks (Supplementary Fig. 5). As shown in Fig. 7a, we find a weak but statistically significant increase in the eigenvalue of the largest functional mode (*r* = 0.16, FDR-corrected *p* = 0.04), suggesting that the pattern of interregional functional interactions is increasingly governed by this leading mode with age. However, no statistically significant increase is observed in the eigenvalues of the second and third functional modes across the lifespan (Fig. 7b; 2nd mode, *r* = −0.07, FDR-corrected *p* = 0.36; 3rd mode, *r* = −0.16, FDR-corrected *p* = 0.04). We further calculate the functional diversity (FD) for each participant, which measures the dispersion of the contribution of different functional modes (see Methods). Larger functional diversity indicates that the pattern of functional interactions is governed to a greater extent by distinct functional modes, whereby the distribution of functional modes’ eigenvalues is closer to a uniform distribution. As shown in Fig. 7c, we observed a statistically significant association between FD and age (*r* = −0.15, *p* = 0.03), indicating age-related decreases in functional diversity across the human lifespan. Taken together, these findings suggest that the diversity of functional modes gradually decreases with age, with the pattern of brain functional interactions increasingly dominated by the most contributing functional mode.

We next examine how structure–function coupling relationships vary with age within the proposed mapping framework. We utilize the structural eigenspectrum to decompose the functional mode that captures the essence of the FC network into two separate components: one is the structure-aligned functional component, which potentially represents direct dependence on physical connections, and the other is the structure-deviated component, which potentially reflects intermediate polysynaptic interactions in the structural network. (Fig. 7d; Methods). The norms of these two components quantify the extent to which functional interactions are organized in an aligned or deviated manner atop the underlying structural connectome. To investigate whether structure and function evolve synergistically or divergently, we introduce a liberal index which is estimated as the energy ratio of structure-aligned and structure-deviated functional components, and then correlate it with the subjects’ ages. As shown in Fig. 7e, we observe a decline in the liberal index (*r* = −0.18, *p* = 0.01), suggesting that the liberality between brain structure and function gradually decreases with age. We further find that age-related alterations in structure–function coupling relationships can be dissociable. We find that the magnitude of functional deviation decreases throughout the lifespan (*r* = − 0.18, *p* = 0.01) whereas variability in functional alignment does not exhibit a statistically significant correlation with age (*r* = 0.08, *p* = 0.39). This indicates that even though both types of coupling patterns contribute to functional interactions, structure-deviated portions are the ones that reflect inter-individual variation during human brain development and aging.

## Discussion

In this work, based on network eigenmodes, we propose a mapping method that allows SC and FC networks to be tightly linked in a simple manner. We first show that the essence of observed FC networks can be characterized by a just few inherent modes, i.e., those with large eigenvalues. This observation demonstrates the utility of eigenmodes for dimensionality reduction, which is key to establishing the proposed mapping method. Typically, the functional mode with the largest eigenvalue is of particular interest in terms of its essential role in governing the formation of functional connectivity. By projecting this functional mode into the parsimonious basis formed by mutually orthogonal structural eigenmodes, we establish a concise and strong mapping between brain structural and functional networks, whose prediction performance is verified on three independent datasets (LAU, NKI, and HCP).

Biologically, by decomposing the intricate functional interactions into a set of functional modes, our approach conceptualizes functional interactions as the aggregation of distinct functional configurations emerging from structural organization. This is consistent with the emerging idea that emphasizes indirect, collective interactions via multiple structural connections^29^. That is, by re-expressing the functional connectivity in terms of functional modes organized atop the underlying anatomical network, our methodology enables us to view neuronal coactivation as an emergent property shaped by the entire structural connectome. This connectome-oriented perspective departs from the traditional perspective that treats pairwise FC as discrete entries, complementing our understanding of brain functional connectivity. For example, we find that although different functional modes constitute an abundant repertoire of brain states, interregional functional connectivity can be well approximated using just a few of them. In other words, interregional functional interactions are dominated by a few principle modes, accompanied by subtle contributions of the remaining multiple modes, and correspondingly possess moderate diversity in terms of functional modes. This heterogeneous distribution of functional mode contributions places the brain in an intermediate state between a specific state governed exclusively by one specific mode and a disordered state induced uniformly by all functional modes, potentially approaching a critical regime where complex dynamics, flexible transitions, and advanced functionality may emerge^51–54^. This also coincides with studies suggesting that the brain shows characteristics of criticality to balance segregated and integrated processing and to maximize dynamical responses^43,55,56^. Moreover, we find the diversity of functional modes exhibits slight but statistically significant decreases with age, with the functional interactions increasingly dominated by one leading functional mode. In light of previous findings that enriched activation patterns are an expected property of functional organization to underlie more efficient processing and information encoding^43,57,58^, we hypothesize that this slight decrease in functional mode diversity might reflect a reorganization of neuronal coactivation patterns, in parallel with a decline in cognition and memory during normal aging. Consistent with this hypothesis, studies on psychedelics identify a variation in the repertoire of active brain patterns under different states of consciousness^59,60^.

In line with previous studies^27,32,33^, we find that the performance of the proposed SC-FC mapping approach is regionally heterogeneous across the brain, with a high correlation between empirical and predicted FC in unimodal regions and a low correlation in transmodal regions. One prominent account posits that rapid expansion of the cerebral mantle may have released cortical organization from strong constraints of primary sensory-motor hierarchies and canonical activity cascades^61^. Association areas fill the gaps between these sensory-motor hierarchies, marked by distributed cortical organization and noncanonical circuit properties^62^. That is, besides the canonical hierarchical form of projections that facilitate progressive feedforward/feedback information flow, the association cortex also possesses parallel and reentrant pathways by which diverse information from multiple functional modalities can be integrated^61,63,64^. Such abundant circuit structure-supporting increasingly diverse spontaneous dynamics and interaction patterns may potentially render the proposed mapping based on the principal functional mode less effective in capturing functional connectivity of transmodal regions. Considering multiple patterns simultaneously may engender better interpretation and deeper understanding, which is an exciting future direction worthy of further investigation.

To explore how structural modes are recruited to form functional connectivity, we exploit the eigendecomposition of the anatomical connectome, which generates a set of orthogonal structural eigenmodes sorted in decreasing order of their eigenvalues. These structural eigenmodes constitute the repertoire of distributed patterns supported by anatomical connectivity, each one associated with a specific spatial frequency, from smooth variation to refined-grained changes. In particular, low-frequency/structure-aligned eigenmodes (i.e., those with positive eigenvalues) exhibit systematical variation along several main geometrical axes (e.g., center-peripheral, anterior-posterior, anterior-middle-posterior, and left-right). Despite the potential switching of order, these spatial patterns are consistently observed in individual subjects and in independent datasets, implying that the hierarchical architecture may be a primary principle of anatomical connections organization. Previous studies have reported systematical hierarchical variation across the cortex in cytoarchitecture^65^, myeloarchitecture^36^, and laminar differentiation^37^. This regional heterogeneity in local attributes may shape how cortical areas are interconnected with each other, resulting in the observed hierarchical patterns of low-frequency eigenmodes. In line with this hypothesis, we find a statistically significant association of these eigenmodes with structural and microstructural gradients, indicating the relevance of interregional connection patterns with hierarchical local properties. Moreover, we find these structural eigenmodes, which are closely aligned with the underlying anatomical connectome, also exhibit significant contributions to functional connectivity formation and display somewhat association with canonical functional systems and functional gradients. These results demonstrate the intertwined relationship between local and global, structural and functional properties, emphasizing the utility of network eigenmodes to bridge the organization of structural and functional connectivity. Furthermore, the high contribution of low-frequency eigenmodes demonstrates the preferential expression of brain activity towards spatially smooth, global patterns on the connectome^32^, implying that the pronounced hierarchical organization of structural modes, stemming from the architecture of anatomical connectivity, may be a key property to support diverse functional interaction patterns. In contrast, high-frequency/structure-deviated eigenmodes (i.e., those with negative eigenvalues) exhibit refined-grained and complex spatial variation, characterizing distributed patterns that are supported by but deviate from the underlying anatomical substrate. The emergence of these structure-deviated modes accords with studies^7^ suggesting that the static structural network could support diverse spatial patterns through collective, network-level interactions, including those decoupled from the physical links.

By decomposing functional modes into structural eigenmodes, our approach differs from the predominant SC-FC coupling analyses^28,31,33^, which quantify SC-FC correspondence by the prediction performance based on structurally derived measures, and differs from the conventional eigenmode approaches^15,16,19^, which assume the eigenmodes of the FC network correspond to those of the SC network (correspond directly or after a rotation operation). Instead, we adhere to an alternative approach to directly disentangle structure-deviated and structure-aligned portions from the functional interaction pattern. These two portions characterize distinct manners in which functional modes are organized atop the anatomical graph, consistent with the intuition that functional interactions must to some extent align with the underlying anatomy due to direct signaling, but also to some extent deviate from this anatomy due to polysynaptic communication. In this way, we could distinguish the respective roles of ‘intermediate polysynaptic interactions’ (deviation) versus simple ‘signaling along the physical connections’ (alignment) by quantifying their intensity. This approach also facilitates the identification of potentially behavior-sensitive or stimulus-sensitive components of functional connectivity, promoting a more refined investigation of individual alterations associated with phenotypes and traits. In the application to the human lifespan, we find that structure–function liberality decreases with age. We also identify the age-sensitive components of functional connectivity–we find that structure-deviated functional components weaken with age whereas the magnitude of structure-aligned components is preserved with age. This observation is particularly interesting in the context of the prior finding that key information for individual identification is found in the functional component deviated from structure^66^, implying that structure–function liberality may be an individually variable feature reflecting the inner workings of the brain. Furthermore, several previous studies have demonstrated correlations between the structure–function coupling relationship and inter-individual variability in cognitive traits^20,67–69^. Stronger structure–function coupling is found to be associated with better abilities of complex cognition, such as reasoning and cognitive switching, which may benefit from reliable and efficient information transmission^20,70^. In contrast, other cognitive traits, such as the level of awareness and attention maintenance, are considered to benefit from less alignment between structure and function, a configuration that might be instrumental for information integration across the brain^25,66^. Combined with these complementary roles of different degrees of structure–function alignment, our findings of age-related decreases in structure-deviated functional components may promote mechanistic insights concerning cognitive changes across the human lifespan, with important implications in inferring physiological processes involved in the aging of brain. Alterations due to cognitive tasks, lesions, and neurological diseases might be another promising application of the proposed approach that would provide valuable insights.

There are several limitations and possible developments in this study. First, while we mainly focus on the most contributing functional eigenmode in the structure–function mapping of brain connectomes, other functional modes also leave their signature on the formation of the FC network. Given the complex and diverse neurobiological mechanisms involved in signaling and synchrony among brain regions, mapping frameworks that incorporate more functional modes have the potential to produce a richer interpretation of how neuronal coactivation patterns emerge from the underlying structural substrate and facilitate a more refined identification of functional components relevant to individual differences in cognitive performance. In addition, our structure–function mapping does not integrate temporal dynamics^71^ or biological details^72^. Thus, another direction for future research is to enrich structure–function mappings with biophysical dynamics, in concert with more nuanced network reconstructions of local attributes, which would promote a more comprehensive understanding of how the brain’s physical wiring supports function.

## Methods

### Data

In this study, we performed the main analyses in two independent datasets. The first one was collected by Department of Radiology, University Hospital Center and University of Lausanne (LAU)^73^. This dataset included 70 healthy participants (27 females, 28.8 ± 9.1 years old). Informed consent approved by the Ethics Committee of Clinical Research of the Faculty of Biology and Medicine, University of Lausanne was obtained from all participants. Diffusion spectrum images (DSI) were acquired on a 3-Tesla magnetic resonance imaging (MRI) scanner (Trio, Siemens Medical, Germany) using a 32-channel head-coil. The protocol was comprised of (1) a magnetization-prepared rapid acquisition gradient echo (MPRAGE) sequence sensitive to white/gray matter contrast (1-mm in-plane resolution, 1.2-mm slice thickness), (2) a DSI sequence (128 diffusion-weighted volumes and a single b0 volume, maximum b-value 8,000 s/mm^2^, 2.2 × 2.2 × 3.0 mm voxel size), and (3) a gradient echo EPI sequence sensitive to blood oxygen level-dependent (BOLD) contrast (3.3-mm in-plane resolution and slice thickness with a 0.3-mm gap, TR 1,920 ms, resulting in 280 images per participant). Gray matter was divided into 68 brain regions following Desikan-Killiany atlas^74^ and further subdivided into 219 approximately equally sized nodes according to the Lausanne anatomical atlas using the method proposed by Cammoun et al.^75^. Individual structural networks were constructed using deterministic streamline tractography, initiating 32 streamline propagations per diffusion direction for each white matter voxel^76^. Functional networks were reconstructed using fMRI data from the same individuals. fMRI volumes were corrected for physiological variables, including regression of white matter, cerebrospinal fluid, and motion. fMRI time series were lowpass filtered. The first four volumes were discarded and motion scrubbing was performed^77^. Individual functional connectivity matrices were constructed by estimating the Pearson correlation between the fMRI time series of each pair of brain regions. A group-average functional connectivity matrix was estimated by averaging all individuals’ functional matrices. Note that one subject was excluded due to missing fMRI data, and therefore 69 subjects were retained for the individual structure–function mappings. More details regarding network construction can be obtained online at the LAU website (https://zenodo.org/record/2872624#.XOJqE99fhmM).

The second one was the Nathan Kline Institute (NKI)/Rockland Sample public dataset^78^, available at http://umcd.humanconnectomeproject.org. This dataset provides preprocessed structural and functional connectome data for 196 participants (82 females, age range = 4–85). Informed consent approved by the Institutional Review Board was obtained from all participants (informed consent was also obtained from child participants and their legal guardians). The scan was performed in a Siemens Trio 3T scanner. The protocol consisted of (1) 10-min resting state fMRI scan (R-fMRI), (2) 6-direction diffusion tensor imaging (DTI) scan, (3) 64-direction diffusion tensor imaging scan (2mm isotropic), (4) MPRAGE anatomical scan, (5) MPRAGE anatomical scan SHORTER sequence, (6) T2 weighted sequence. A comprehensive description of data acquisition is available at the NKI website (http://fcon_1000.projects.nitrc.org/indi/pro/nki.html). For fMRI data, the preprocessing procedure includes slice timing correction, rigid-body motion correction, spatially smooth with 5mm FWHM Gaussian, scale to mean 10000, band-pass filtering from 0.08 to 0.009Hz, brain tissue segmentation, nuisance regression (mean CSF, mean WM, whole brain signal, the six motion parameters, and all temporal derivatives), motion scrubbing (relative motion displacement > 0.5 mm or relative BOLD signal intensity change > 0.5%)^77^, and registration. The Craddock 200 atlas^79^ is applied to fMRI data to derive 188-ROI parcellation. All subjects were retained as no one had more than 100 TRs flagged by motion scrubbing. For DTI data, the preprocessing procedure includes corrections of motion and eddy current distortions, diffusion tensor estimation, tractography using the fiber assignment by continuous tracking (FACT) algorithm^80^, and registered fractional anisotropy (FA) map to the MNI152 average brain. The Craddock 200 atlas^79^ was registered to each individual’s DTI space to construct structural connectivity. A more detailed description is provided by Brown et al.^81^.

Additionally, we introduced the third dataset from the Human Connectome Project (HCP) (HCP 100 Unrelated Subjects dataset)^49^ to assess the out-sample performance of distinct mapping methods. Informed consent approved by the Washington University Institutional Review Board was obtained from all participants. All acquisitions were first preprocessed according to HCP-minimal preprocessing guidelines^82^. Then a bandpass filtering between 0.01Hz and 0.08Hz was applied to functional data. Individual structural networks were derived from the preprocessed diffusion MRI data using the MRtrix3 software [http://www.mrtrix.org/]. The detained operations included the estimation of multi-tissue response function^83^, the estimation of fiber orientation distributions using the multi-shell multi-tissue constrained spherical deconvolution^84^, the generation of a whole-brain tractogram with 5 million streamlines using a probabilistic approach (iFOD2)^85^ and the anatomically constrained tractography (ACT)^86^ algorithm with dynamic seeding, the estimation of tract weights (SIFT2)^87^ to reduce reconstruction biases. Finally, the tractogram was mapped onto the Schaefer400 atlas^88^ to create a structural connectome. The structural connectivity between pairs of regions was further scaled by the inverse of two region volumes^89^. The resting state fMRI data from each subject contains two sessions, each of which contains two scans with opposite phase encoding directions (left-to-right and right-to-left encodings). Here, we make use of the first session to learn model parameters and the second one to evaluate mapping performance. fMRI data were parcellated according to the same atlas used for structural networks. We concatenated the two acquisitions to produce a single functional connectivity matrix per session. Functional connectivity is constructed by estimating the Pearson correlation between the fMRI time series of each pair of brain regions. For more details regarding the acquisition protocol and minimal preprocessing see refs. ^49^ and ^82^. We removed individuals with high motion (mean framewise displacement > 0.25mm or max framewise displacement > 2mm)^77^, and finally retained 78 subjects for the subsequent analysis. All ethical regulations relevant to human research participants were followed.

### Structural and functional modes

Applying an eigendecomposition, the FC network can be decomposed as

1 $${{{{{{{\bf{FC}}}}}}}}={{{{{{{\bf{U}}}}}}}}{\Lambda }^{f}{{{{{{{{\bf{U}}}}}}}}}^{T},$$

where the eigenvalues are represented by ${\Lambda }^{f}={\{{\lambda }_{i}^{f}\}}_{1\le i\le N}$ and eigenvectors are represented by ${{{{{{{\bf{U}}}}}}}}={\{{{{{{{{{\bf{U}}}}}}}}}_{i}\}}_{1\le i\le N}$. N indicates the number of network nodes. Several negative eigenvalues that may be induced by the noise were set to 0, which does not result in significant losses of information on FC networks^43^. According to the spectral graph theory^42^, these mutually orthogonal eigenvectors **U** can be interpreted as the N inherent constituent modes of the FC network. As all eigenmodes are scaled to the unit norm, the magnitude of eigenvalues mirrors the contribution of the corresponding functional mode to the FC network. Typically, the eigenmode with the largest eigenvalue represents the functional pattern that has the greatest impact on the formation of functional connectivity. Conversely, eigenmodes with zero eigenvalues are considered to have no contribution to FC, i.e. the FC network does not possess that functional mode. The alternative expression is

2 $${{{{{{{\bf{FC}}}}}}}}=\mathop{\sum }\limits_{i}^{N}{{{{{{{{\bf{U}}}}}}}}}_{i}{\lambda }_{i}^{f}{{{{{{{{\bf{U}}}}}}}}}_{i}^{T},$$

representing the FC network as the linear superposition of *N*-independent functional modes.

Similarly, the SC network can be decomposed as

3 $${{{{{{{\bf{SC}}}}}}}}={{{{{{{\bf{V}}}}}}}}{\Lambda }^{s}{{{{{{{{\bf{V}}}}}}}}}^{T},$$

with the eigenvalues ${\Lambda }^{s}={\{{\lambda }_{j}^{s}\}}_{1\le j\le N}$ quantifying the smoothness (alignment) of the inherent modes specified by eigenvectors ${{{{{{{\bf{V}}}}}}}}={\{{{{{{{{{\bf{V}}}}}}}}}_{j}\}}_{1\le j\le N}$. Specifically, The alignment of eigenmodes indicates to what degree the spatial patterns of structural eigenmodes are aligned with or deviated from the underlying structural network. To capture this intuition, we generalize the basic functions (sinusoids) with different temporal frequencies in the classical Fourier transform to the structural eigenmodes which can be viewed as basic modes with different spatial frequencies in the Graph Fourier Transform (Supplementary Fig. 8). In the time domain, the low-frequency temporal signal varies slowly along the time dimension (i.e., data points that are close in time have similar values) whereas the high-frequency temporal signal changes fast over time (i.e., data points may have very dissimilar values even if they are at adjacent time moment). In the graph domain, low-frequency/structure-aligned eigenmodes vary smoothly across the graph (i.e., nodes that are tightly connected tend to have similar values) whereas high-frequency/structure-deviated eigenmodes exhibit fine-grained variations across the graph (i.e., nodes may have very different values even if they are adjacent in the graph). Thus, just as the temporal frequency of signals reflects their dependence on time, the alignment of structural eigenmodes reflects the degree to which they are constrained by the underlying anatomical connectome. Mathematically, the alignment of the *j*-th structural eigenmodes^20^ can be expressed by

4 $$\mathop{\sum }\limits_{{n}_{1},{n}_{2}=1}^{N}{{{{{{{\bf{SC}}}}}}}}({n}_{1},{n}_{2}){{{{{{{{\bf{V}}}}}}}}}_{j}({n}_{1}){{{{{{{{\bf{V}}}}}}}}}_{j}({n}_{2})={{{{{{{{\bf{V}}}}}}}}}_{j}^{T}{{{{{{{\bf{SC}}}}}}}}{{{{{{{{\bf{V}}}}}}}}}_{j}.$$

In this expression, *n*_1_, *n*_2_ represent network nodes and **SC**(*n*_1_, *n*_2_) = 0 for *n*_1_ = *n*_2_. Note that ${{{{{{{{\bf{V}}}}}}}}}_{j}^{T}{{{{{{{\bf{SC}}}}}}}}{{{{{{{{\bf{V}}}}}}}}}_{j}={\lambda }_{j}^{s}$ is the *j*-th eigenvalue, a quantity that will be positive if the structural mode **V**_*j*_ is aligned to the underlying connectivity (values of most connected nodes possess same signs) and will be negative if the mode **V**_*j*_ deviates from the connectivity (values of most connected nodes possess different signs). By sorting structural eigenvectors in descending order of their eigenvalues, we construct an eigenspectrum spanning from structural modes closely aligned with SC to modes deviated from SC. In the “Results” section, to understand how the alignment of these structural eigenmodes is informed by connections of different distances, we divide structural connections into 10 distance bins in ascending order of their lengths (equal number of connections in each bin) and measure the alignment of SC eigenmodes with respect to structural connections contained within each bin. Specifically, for a given eigenmode **V**_*j*_ and distance bin k, the alignment was quantified by ${\sum }_{e({n}_{1},{n}_{2})\in bi{n}_{k}}{{{{{{{\bf{SC}}}}}}}}({n}_{1},{n}_{2}){{{{{{{{\bf{V}}}}}}}}}_{j}({n}_{1}){{{{{{{{\bf{V}}}}}}}}}_{j}({n}_{2})$, where *e*(*n*_1_, *n*_2_) ∈ *bin*_*k*_ indicates the set of connections within the *k*-th bin.

### SC-FC mapping

The link between brain structural and functional networks can be constructed by projecting functional modes ${\{{{{{{{{{\bf{U}}}}}}}}}_{i}\}}_{1\le i\le N}$ into structural modes ${\{{{{{{{{{\bf{V}}}}}}}}}_{j}\}}_{1\le j\le N}$:

5 $${{{{{{{{\bf{U}}}}}}}}}_{i}={m}_{i1}{{{{{{{{\bf{V}}}}}}}}}_{1}+{m}_{i2}{{{{{{{{\bf{V}}}}}}}}}_{2}+\cdots +{m}_{iN}{{{{{{{{\bf{V}}}}}}}}}_{N}=\mathop{\sum }\limits_{j=1}^{N}{m}_{ij}{{{{{{{{\bf{V}}}}}}}}}_{j},$$

where parameters ${\{{m}_{ij}\}}_{1\le i,j\le N}$ can be computed as ${m}_{ij}={{{{{{{{\bf{V}}}}}}}}}_{j}^{T}{{{{{{{{\bf{U}}}}}}}}}_{i}$. Note that for any *i*, we have

6 $$\mathop{\sum }\limits_{j=1}^{N}{({m}_{ij})}^{2}=\mathop{\sum }\limits_{j=1}^{N}{m}_{ij}{\left({{{{{{{{\bf{V}}}}}}}}}_{j}^{T}{{{{{{{{\bf{U}}}}}}}}}_{i}\right)}^{T}={{{{{{{{\bf{U}}}}}}}}}_{i}^{T}\left(\mathop{\sum }\limits_{j=1}^{N}{m}_{ij}{{{{{{{{\bf{V}}}}}}}}}_{j}\right)={{{{{{{{\bf{U}}}}}}}}}_{i}^{T}{{{{{{{{\bf{U}}}}}}}}}_{i}=1.$$

The magnitude of ${m}_{ij}^{2}$ can thus be used to reflect the contribution of structural mode **V**_*j*_ to functional mode **U**_*i*_^43^.

The FC network can be represented as

7 $${{{{{{{\bf{FC}}}}}}}} 	 =\mathop{\sum }\limits_{i=1}^{N}{{{{{{{{\bf{U}}}}}}}}}_{i}{\lambda }_{i}^{f}{{{{{{{{\bf{U}}}}}}}}}_{i}^{T}\\ 	 =\mathop{\sum }\limits_{i=1}^{N}{\lambda }_{i}^{f}\left({m}_{i1}{{{{{{{{\bf{V}}}}}}}}}_{1}+\cdots +{m}_{iN}{{{{{{{{\bf{V}}}}}}}}}_{N}\right)\left({m}_{i1}{{{{{{{{\bf{V}}}}}}}}}_{1}^{T}+\cdots +{m}_{iN}{{{{{{{{\bf{V}}}}}}}}}_{N}^{T}\right)\\ 	 =\mathop{\sum }\limits_{i,{j}_{1},{j}_{2} =1}^{N}{\lambda }_{i}^{f}{m}_{i{j}_{1}}{m}_{i{j}_{2}}{{{{{{{{\bf{V}}}}}}}}}_{{j}_{1}}{{{{{{{{\bf{V}}}}}}}}}_{{j}_{2}}^{T}\\ 	 =\mathop{\sum }\limits_{{j}_{1},{j}_{2}=1}^{N}\left(\mathop{\sum }\limits_{i=1}^{N}{\lambda }_{i}^{f}{m}_{i{j}_{1}}{m}_{i{j}_{2}}\right){{{{{{{{\bf{V}}}}}}}}}_{{j}_{1}}{{{{{{{{\bf{V}}}}}}}}}_{{j}_{2}}^{T},$$

Here, we only keep the functional mode with the largest eigenvalue given its essential role in governing the formation of functional connectivity. The estimation of the FC network is then simplified as

8 $${{{{{{{\bf{FC}}}}}}}}\approx {\lambda }_{1}^{f}{{{{{{{{\bf{U}}}}}}}}}_{1}{{{{{{{{\bf{U}}}}}}}}}_{1}^{T}=\mathop{\sum }\limits_{{j}_{1},{j}_{2}=1}^{N}({\lambda }_{1}^{f}{m}_{1{j}_{1}}{m}_{1{j}_{2}}){{{{{{{{\bf{V}}}}}}}}}_{{j}_{1}}{{{{{{{{\bf{V}}}}}}}}}_{{j}_{2}}^{T},$$

where parameters ${\{{m}_{1j}\}}_{1\le j\le N}$ were estimated as ${m}_{1j}={{{{{{{{\bf{V}}}}}}}}}_{j}^{T}{{{{{{{{\bf{U}}}}}}}}}_{1}$. Note that though we focus on the largest functional mode here, future work could naturally incorporate more functional modes in SC-FC mapping to obtain a more refined quantification of the relationship between structure and function (for more details see Supplementary Note 1: SC-FC mapping based on the first K functional modes).

### Performance evaluation

We assess the mapping performance by evaluating Pearson *R* between the upper triangular entries (excluding the diagonal entries) of the predicted and empirical FC matrices. For individuals in LAU and NKI datasets, we perform in-sample evaluation, that is, the mapping performance is evaluated on the same empirical data used to learn model parameters. For individuals in the HCP dataset, we conduct the out-of-ample evaluation, where model parameters are learned using the first resting-state session and evaluated using the second resting-state session.

### Benchmark comparisons

The first one is the eigenmode approach of Tewarie et al.^16^, which approximates the empirical FC network with a weighted linear combination of structural eigenmodes:

9 $${{{{{{{\bf{FC}}}}}}}}\approx {{{{{{{\bf{V}}}}}}}}{{{{{{{\bf{A}}}}}}}}{{{{{{{{\bf{V}}}}}}}}}^{T}=\mathop{\sum }\limits_{j=1}^{N}{a}_{j}{{{{{{{{\bf{V}}}}}}}}}_{j}{{{{{{{{\bf{V}}}}}}}}}_{j}^{T},$$

where **A** is a diagonal matrix, with entries *a*_*j*_ indicating weighting coefficients that can be fitted from empirical data. Previous work has demonstrated that this approach could be used to explain frequency-specific functional networks^90^. Here, we exploit it as a primary benchmark for our approach as it permits a straight comparison between diagonal and non-diagonal projection predictions. By projecting the first functional eigenmode on the structural eigenspectrum, our method contains both the diagonal terms ${\{{{{{{{{{\bf{V}}}}}}}}}_{{j}_{1}}{{{{{{{{\bf{V}}}}}}}}}_{{j}_{2}}^{T}\}}_{{j}_{1} = {j}_{2}}$ (which are also contained in the Tewarie et al.) and the non-diagonal terms ${\{{{{{{{{{\bf{V}}}}}}}}}_{{j}_{1}}{{{{{{{{\bf{V}}}}}}}}}_{{j}_{2}}^{T}\}}_{{j}_{1}\ne {j}_{2}}$ (which may provide additional information in SC-FC mapping). The comparison between these two approaches allows us to examine whether non-diagonal interactions among structural eigenmodes could enhance coupling between the structure and function connectomes. Of note, both methods use the structural eigenspectrum ${\{{{{{{{{{\bf{V}}}}}}}}}_{j}\}}_{1\le j\le N}$ as input and have almost the same number of parameters (${\{{a}_{j}\}}_{1\le j\le N}$ vs ${\{{m}_{1j}\}}_{1\le j\le N}$), thereby enabling a direct comparison of SC-FC mapping strategies without the interference of model complexity (as the increased model complexity generally yields increased explanatory power). In this way, we would like to ascribe the difference between mapping performance of the proposed method and Tewarie et al.^16^ to the introduction of non-linear interactions between different structural eigenmodes.

The second benchmark method is the communication model^14^, which approximates the FC network with a weighted superposition of communication events over the structural network, with the forms of communication ranging from the shortest path routing (centralized) to signal diffusion (decentralized):

10 $${{{{{{{\bf{FC}}}}}}}}\approx {a}_{0}+\mathop{\sum }\limits_{q=1}^{Q}{a}_{q}{{{{{{{{\bf{P}}}}}}}}}_{q},$$

where {*a*_0_, *a*_1_, ⋯  , *a*_*Q*_} are weighting coefficients that can be fitted from empirical data. Predictors ${\{{{{{{{{{\bf{P}}}}}}}}}_{q}\}}_{1\le q\le Q}$ indicate fully weighted matrices derived from the SC data, including flow graphs (parameterized at different timescales)^91^, mean first passage times^92^, communicability^93,94^, matching index^95^, shortest path length, path transitivity (parameterized at weight-to-cost transformations)^13^, search information (parameterized at weight-to-cost transformations)^96^, and Euclidean distance. Specifically, the flow graph is derived from the Markovian process embedded into the weighted structural connections, with the elements corresponding to the probabilistic flow of random walkers at time *t* (here we set *t* = 1,2.5,5,10). The mean first passage time indicates the expected time (the number of steps) a random walker takes to reach a target node for the first time and we convert the values to z-scores for each column to exclude nodal biases. Communicability is defined as a weighted sum of all possible walks between brain regions, which can be calculated by *exp*(**SC**). The matching index measures the similarity of pairwise nodes based on the number of their common neighbors, and the element in this matrix is quantified as $\frac{| {\Gamma }_{{n}_{1}\backslash {n}_{2}}\bigcap {\Gamma }_{{n}_{2}\backslash {n}_{1}}| }{| {\Gamma }_{{n}_{1}\backslash {n}_{2}}\bigcup {\Gamma }_{{n}_{2}\backslash {n}_{1}}| }$, where ${\Gamma }_{{n}_{1}\backslash {n}_{2}}$ represents the neighbors of node *n*_1_ excluding the node *n*_2_. The shortest path length measures the minimum sum of costs to reach the target node from a reference node. Here we use the transformation **SC**^−*γ*^ (*γ* = 0.25, 0.5, 1, 2) to convert edge weights to costs. The path transitivity captures the density of local detours by which walkers departing from the shortest path could reaccess it. Search information measures the amount of information requisite to access the shortest path. These two kinds of predictors are derived the following^13,28^ with the weight-to-cost transformation **SC**^−*γ*^ (*γ* = 0.25, 0.5, 1, 2). Euclidean distance indicates the Euclidean distance between pairwise brain nodes.

The third benchmark method is the spectral mapping of Becker et al.^48^, which approximates the empirical FC network based on a polynomial expansion and a rotation matrix:

11 $${{{{{{{\bf{FC}}}}}}}}\approx {{{{{{{\bf{R}}}}}}}}\left(\mathop{\sum }\limits_{l=0}^{L}{a}_{l}{{{{{{{{\bf{SC}}}}}}}}}^{l}\right){{{{{{{{\bf{R}}}}}}}}}^{T},$$

where ${\{{a}_{l}\}}_{0\le l\le L}$ are weighting coefficients that can be fitted from empirical data. The matrix **R**, which corresponds to a rotation operation to align structural eigenmodes to functional eigenmodes, can be estimated by **UV**^*T*^ for individual mappings. The value of L, which corresponds to the maximum length of the structural walks under consideration, is selected empirically. Here, we conduct this method with varying values of free parameter L and exploit it as a benchmark for the proposed method with varying values of free parameter K (that is, the first K functional eigenmodes under consideration). In fact, our method is somewhat akin to Becker et al.^48^: both approaches attempt to construct a link between structural and functional eigenmodes and the weight matrix **M** of our method can be built from the rotation matrix **R** of Becker et al.^48^ via a suitable transformation **M** = **V**^*T*^**RV**. Thus, it is not a surprise that these two methods yield comparably competitive performance. However, there exist two key differences, with more conciseness and better interpretability offered by our method in contrast to Becker et al.^48^. First, Becker et al. approximates FC with a polynomial expansion of order L whereas our method uses the first K highly contributing functional mode. In other words, Becker et al. seek to explain interregional functional interactions in terms of a weighted superposition of structural walks with different lengths whereas our method aims to capture the essential patterns of functional interactions in terms of a few dominant modes. This change leads to a dramatic simplification of network representation, with the complexity of the mapping method reduced from *O*(*N*^2^) to *O*(*N*). Second, Becker et al. performs a rotation operation (**U** = **RV**), which can be viewed as a change of coordinates to align the eigenmodes of structural and functional connectivity, whereas our method performs a decomposition operation (**U** = **VM**), which permits the identification of different structural eigenmodes’ contributions to functional modes. This change enables the eigenspectrum of the structural network to inform how functional interaction patterns align to or deviate from the underlying white matter connections, promoting a more refined investigation of structure–function relationships. Using our methodology, we are able to disentangle structure-deviated and structure-aligned portions from the dominant functional interaction pattern, which characterize distinct manners in which functional modes are organized atop the anatomical graph. This is interesting because we might assume that functional interactions must to some extent depend on underlying anatomy that supports direct signaling between brain regions, but on the other hand some functional interactions may deviate from this anatomy via polysynaptic communication.

Finally, we introduce a reference mapping that always returns the group-average functional connectivity matrix to assess whether individual mappings that utilize subject-specific structural information could capture additional information not explained by the mean. We perform this reference mapping for individuals from two independent datasets (LAU and NKI). For each dataset, we construct the group-average functional connectivity matrix by averaging all individual subjects’ FC matrices and quantify the performance of this reference mapping by the correlation between the group-average and individual matrices. We then consider it as a baseline and conduct a paired t-test to examine whether individual mappings containing structural inputs (the proposed method, Tewarie et al.^16^, and the communication model^14^) could outperform this mean mapping.

### Functional diversity

To measure the diversity of contributions of different functional modes, here called functional diversity (FD), we estimated the extent to which the distribution of functional eigenvalues is similar to a uniform distribution:

12 $$FD=1-\frac{1}{{N}_{M}}\mathop{\sum }\limits_{i=1}^{M}| \frac{{\lambda }_{i}^{f}}{{\sum }_{i}{\lambda }_{i}^{f}}-\frac{1}{M}| ,$$

where M is the number of functional modes that the FC network possesses and *N*_*M*_ = 2(*M* − 1)/*M* is a normalization factor that restricts the FD to the interval [0,1]. At one extreme where the FD value equals 0, the FC network is completely governed by one inherent mode; at the other extreme where the FD = 1, all functional modes contribute equally to the formation of functional interactions.

### Structure–function liberality

Within the present analytical framework, the inherent pattern of functional interactions can be investigated in the context of a structural eigenspectrum spanning from modes closely aligned to anatomical connections (those with positive structural eigenvalues) to modes deviated from the anatomy (those with negative structural eigenvalues). Here, we exploit the Graph Fourier Transform (GFT)^42^ and spectral filtering to split the most contributing functional mode into two separate components: one represented by the first *L*_*A*_ structural modes, exhibiting tight coupling with the structure, and the other represented by the last *L*_*D*_ structural modes, exhibiting flexible deviations from the structural substrate. That is,

13 $${{{{{{{{\bf{U}}}}}}}}}_{1}^{A}={m}_{11}{{{{{{{{\bf{V}}}}}}}}}_{1}+{m}_{12}{{{{{{{{\bf{V}}}}}}}}}_{2}+\cdots +{m}_{1{L}_{A}}{{{{{{{{\bf{V}}}}}}}}}_{{L}_{A}},$$

14 $${{{{{{{{\bf{U}}}}}}}}}_{1}^{D}={m}_{1N-{L}_{D}+1}{{{{{{{{\bf{V}}}}}}}}}_{N-{L}_{D}+1}+\cdots +{m}_{1N}{{{{{{{{\bf{V}}}}}}}}}_{N},$$

where ${{{{{{{{\bf{U}}}}}}}}}_{1}^{A}$ and ${{{{{{{{\bf{U}}}}}}}}}_{1}^{D}$ denote structure-aligned and structure-deviated components of the functional mode, respectively. ${\{{m}_{1j}\}}_{1\le j\le N}$ are parameters estimated in SC-FC mapping procedure. Considering that there is no general method to determine the threshold *L*_*A*_ and *L*_*D*_, we chose a default value (*L*_*A*_ = *L*_*D*_ = 10) following the previous literature^20^ and performed a sensitivity analysis to confirm the robustness of results to threshold selection (Supplementary Fig. 9). The intensity of the aligned and deviated portions was measured as the norms of ${{{{{{{{\bf{U}}}}}}}}}_{1}^{A}$ and ${{{{{{{{\bf{U}}}}}}}}}_{1}^{D}$. We further introduce the structure–function liberality, which is estimated by the energy ratio between the structure-aligned and structure-deviated components, to identify to what degree the functional interaction pattern is misaligned versus aligned with the structure. Correlating this liberal index with age, we could explore the evolving property of structure–function relationships across the human lifespan. There existed two distinct possibilities: (1) the structure–function liberality was preserved with age; (2) the structure–function liberality exhibited age-related change. The first one indicates that the structure–function relationship is preserved with age, implying that lifespan differences in FC networks may be simply induced by changes in structural architecture. The second one suggests that SC and FC networks change divergently with age, with increasing or decreasing liberality indicating that functional interaction patterns are gradually untethered or tethered by structural constraints.

### Statistics and reproducibility

Spatially constrained permutation tests (spin tests, 10,000 permutations) are performed to examine the statistical significance of spatial analyses, including the spatial similarities of the first four SC eigenmodes to structural and functional organization features as well as the effects of resting-state networks on regional structure–function coupling. The *p*-values are calculated as the proportion of simulated test statistics that are more extreme than the observed test statistic and are corrected for multiple comparisons. Paired *t* tests are performed to test the statistically significant differences between the prediction performance of distinct approaches. The correlation values are calculated by Pearson R. Results are replicated using the LAU (*n* = 69 subjects), NKI (*n* = 196 subjects), and HCP (*n* = 78 subjects) datasets and using different structure-aligned and structure-deviated thresholds.

### Reporting summary

Further information on research design is available in the Nature Portfolio Reporting Summary linked to this article.

### Supplementary information

Supplementary Information

Description of Additional Supplementary Data

Supplementary Data 1

Reporting Summary

## Data Availability

The Lausanne dataset is publicly available at https://zenodo.org/record/2872624#.XOJqE99fhmM. The Nathan Kline Institute (NKI)/Rockland Sample public dataset is publicly available at http://fcon_1000.projects.nitrc.org/indi/pro/nki.html. The HCP 100 dataset is available at https://www.humanconnectome.org/study/hcp-young-adult. Source data for Figs. 2–7 and Supplementary Figs. 1–7, 9 are provided in Supplementary Data 1.

## Code availability

The structural connectomes for HCP are constructed through the MRtrix3 software [http://www.mrtrix.org/]. The eigendecomposition of SC and FC networks is implemented using the function eig.m in Matlab R2020a. Code for the spin test is publicly available at https://github.com/spin-test/spin-test.
