# Supplementary material for: Eigenmode-based approach reveals a decline in brain structure–function liberality across the human lifespan
Source: Commun Biol. 2023 Nov 7;6:1128. doi: 10.1038/s42003-023-05497-4 (PMC10630517; doi:10.1038/s42003-023-05497-4)
Supplement: Supplementary file 1 — Supplementary Information [file 42003_2023_5497_MOESM1_ESM.pdf]

Eigenmode-based approach reveals a decline in brain structure-function liberality across the human lifespan

Yaqian Yang, Shaoting Tang*, Xin Wang*, Yi Zhen, Yi Zheng,
Hongwei Zheng, Longzhao Liu, Zhiming Zheng

*To whom correspondence should be addressed;
E-mail: tangshaoting@buaa.edu.cn; wangxin_1993@buaa.edu.cn

Supplementary Information

Supplementary Note 1: SC-FC mapping based on the first K functional modes

In this section, we seek to expand our mapping with the first K functional modes under consideration. Specifically, we decompose the FC network as $\mathbf{FC} = \mathbf{U}\boldsymbol{\Lambda}^f\mathbf{U}^T$ with the functional eigenvalues $\boldsymbol{\Lambda}^f = \{\lambda_i^f\}_{1\leq i\leq N}$ and functional eigenmodes $\mathbf{U} = \{\mathbf{U}_i\}_{1\leq i\leq N}$ sorted in a descending order of eigenvalues. These functional eigenmodes represent intrinsic constituent modes and the magnitude of eigenvalues informs the contribution of the corresponding functional mode to the FC network [1, 2]. Typically, functional modes with large eigenvalues are of particular interest due to their dominant role in capturing functional interaction patterns. Similarly, the SC matrix can be decomposed as $\mathbf{SC} = \mathbf{V}\boldsymbol{\Lambda}^s\mathbf{V}^T$, in which structural eigenvalues $\boldsymbol{\Lambda}^s = \{\lambda_j^s\}_{1\leq j\leq N}$ and structural eigenvectors $\mathbf{V} = \{\mathbf{V}_j\}_{1\leq j\leq N}$ are sorted in a descending order of eigenvalues. These N mutually orthogonal structural eigenmodes form a basis set into which any of the functional modes can be decomposed:

$$\mathbf{U}_i = m_{i1}\mathbf{V}_1 + m_{i2}\mathbf{V}_2 + \cdots + m_{iN}\mathbf{V}_N = \sum_{j=1}^{N} m_{ij}\mathbf{V}_j, \tag{1}$$

where $\mathbf{M}$ is a weight matrix with elements $m_{ij} = \mathbf{V}_j^T\mathbf{U}_i$. The mapping between structural and functional connectivity networks based on the first K functional modes can thus be expressed as:

$$\mathbf{FC} \approx \sum_{i=1}^{K} \lambda_i^f \mathbf{U}_i\mathbf{U}_i^T = \sum_{j_1,j_2=1}^{N} (\sum_{i=1}^{K} \lambda_i^f m_{ij_1} m_{ij_2})\mathbf{V}_{j_1}\mathbf{V}_{j_2}^T. \tag{2}$$

The value of K, which sets the degrees of freedom of the model to $KN + K$, is empirically selected to tune the balance of prediction accuracy and computational complexity according to the tasks. Here, we gradually increase the value of K in individual SC-FC mappings for subjects from the LAU and NKI datasets and quantify the performance by Pearson correlation between the upper-triangular part (excluding diagonal elements) of the predicted and empirical FC matrices. Supplementary Figure 5 illustrates the results of estimated functional connectivity

networks using our method with K ranging from 1 to 3. One can note that the mapping performance consistently improves with an increasing number of functional modes under consideration. We then compare the mapping performance of our proposed method against the work from Becker et al. [3] that approximates the FC matrix based on a polynomial expansion of the SC matrix and a rotation matrix. Figure 6 illustrates the performance of the proposed mapping over different values of K (the number of functional modes under consideration) and the results of Becker et al. over different values of L (the maximum length of the structural walks). We find that, irrespective of model complexity, both the proposed method and the Becker et al. [3] have competitive SC-FC mapping performance (e.g., proposed: $R = 0.97 \pm 0.01$ when $K = 8$; Becker et al: $R = 0.99 \pm 0.00$ when $L = 8$; LAU). We further examine the computation time of these two approaches. We run the codes on a Windows desktop: Intel Core i7-6700, 3.40GHz, 4 cores, 8 logical processors, 16GB of memory. We use MATLAB R2020a and apply both approaches to the HCP dataset. We find that the proposed approach has a runtime of 0.2s per subject ($K = 10$), shorter than Becker et al (0.9s per subject; $L = 10$). Finally, we compare our approach with the previous work [4] based on Riemannian manifold optimization using the HCP dataset. The hyperparameter values of the Riemannian approach are selected following the study [4]. Note that the Riemannian approach does not converge for 8 subjects and the results are reported for the remaining 70 subjects. We hypothesize this is because the Riemannian approach is intended for multi-subject mapping (that is, the strategy to find a common mapping between structure and function for all subjects simultaneously), rather than the single-subject mappings (that is, the strategy to find a specific mapping for an individual subject). Supplementary Figure 7 illustrates the performance of the proposed mapping with K from 1 to 10 and the results of the Riemannian approach with the walk length from 1 to 10. We find that both approaches achieve competitive performance as the values of K or walk length increased (e.g., the proposed: $R = 0.760.06$ for $K = 10$; Riemannian: $R = 0.760.06$ for walk length=10).

Supplementary References

[1] Piet Van Mieghem. *Graph spectra for complex networks*. Cambridge University Press, 2010.

[2] Dongya Wu, Lingzhong Fan, Ming Song, Haiyan Wang, Congying Chu, Shan Yu, and Tianzi Jiang. Hierarchy of connectivity–function relationship of the human cortex revealed through predicting activity across functional domains. *Cerebral Cortex*, 30(8):4607–4616, 2020.

[3] Cassiano O Becker, Sérgio Pequito, George J Pappas, Michael B Miller, Scott T Grafton, Danielle S Bassett, and Victor M Preciado. Spectral mapping of brain functional connectivity from diffusion imaging. *Scientific reports*, 8(1):1411, 2018.

[4] Oualid Benkarim, Casey Paquola, Bo-yong Park, Jessica Royer, Raúl Rodríguez-Cruces, Reinder Vos de Wael, Bratislav Misic, Gemma Piella, and Boris C Bernhardt. A riemannian approach to predicting brain function from the structural connectome. *NeuroImage*, 257:119299, 2022.

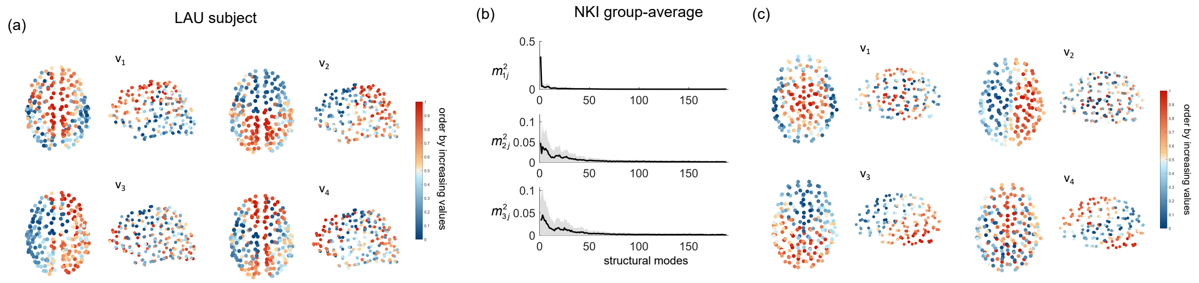

Supplementary Figure 1. Highly-contributing structural eigenmodes for a representative subject in LAU dataset and for subjects in NKI dataset. (**a**) The spatial distribution of the first four structural eigenmodes derived from an individual subject in LAU dataset. (**b**) The contribution of individual structural eigenmodes to the first three functional eigenmodes for all subjects (gray lines) in NKI dataset, with the black line showing the mean value across subjects (n=196 subjects). (**c**) The spatial distribution of the first four structural eigenmodes derived from the group-average structural connectome in NKI dataset.

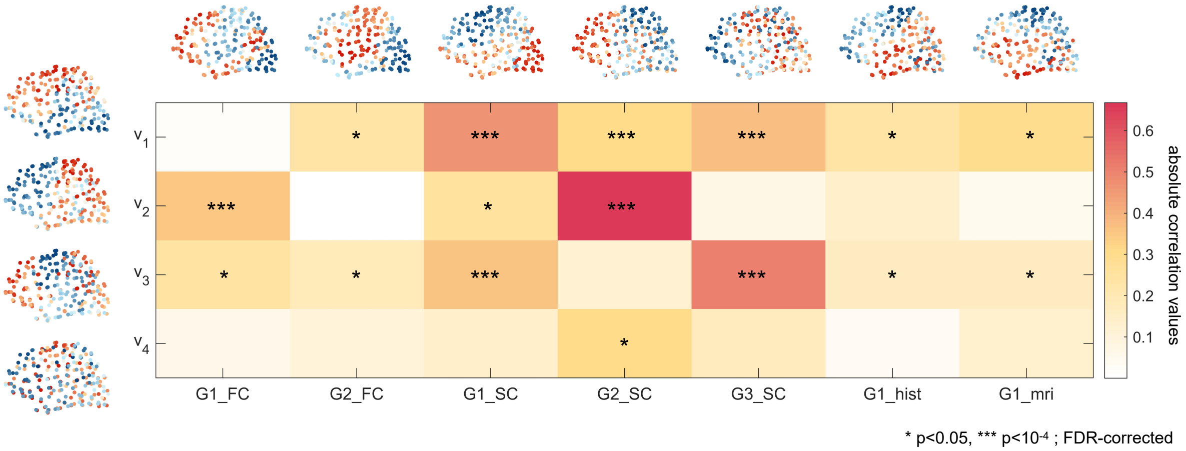

Supplementary Figure 2. Association between structural eigenmodes and structural/functional features. v_1, v_2, v_3, and v_4 indicate the first four structural eigenmodes derived from the group-average structural connectome in LAU dataset. G1_FC and G2_FC indicate the first two functional gradients (G1_FC: unimodal-transmodal; G2_FC: visual-motor). G1_SC, G2_SC, and G3_SC indicate the first three structural gradients (G1_SC: inferior-superior; G2_SC: anterior-posterior; G3_SC: medial-lateral). G1_hist and G1_mri indicate microstructural gradients (G1_hist: sensory/motor-transmodal/limbic; G1_mri: primary sensory-limbic). * indicates $p < 0.05$, *** indicates $p < 10^{-4}$, FDR-corrected.

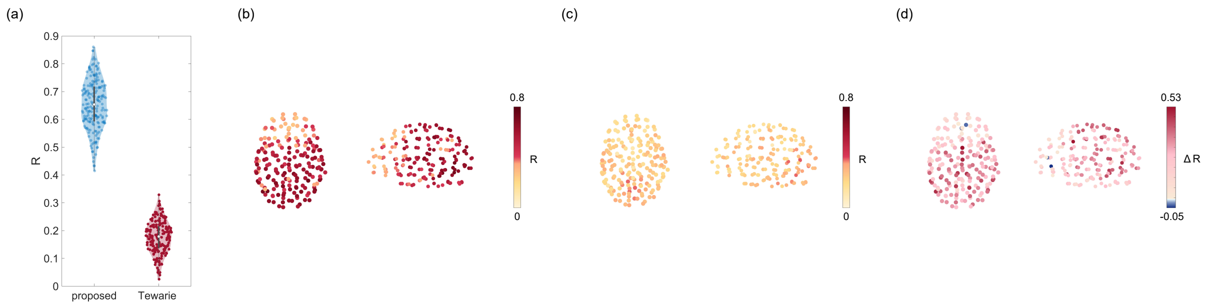

Supplementary Figure 3. The performance of the proposed model in NKI dataset. (**a**) The correlation R of the proposed method vs. the eigenmode approach of Tewarie et al. for individual subjects in NKI dataset (n=196 subjects). In each violin plot, the box indicates the interquartile range and the empty circle indicates the median value. Specifically, $R = 0.65 \pm 0.09$ for the proposed approach and $R = 0.18 \pm 0.06$ for Tewarie et al. (**b**) The spatial pattern of SC-FC coupling estimated by the proposed method. (**c**) The spatial pattern of SC-FC coupling estimated by Tewarie et al. (**d**) Regional differences in R values of the proposed mapping and Tewarie et al. $77 \pm 8\%$ of brain regions are better explained by the proposed mapping.

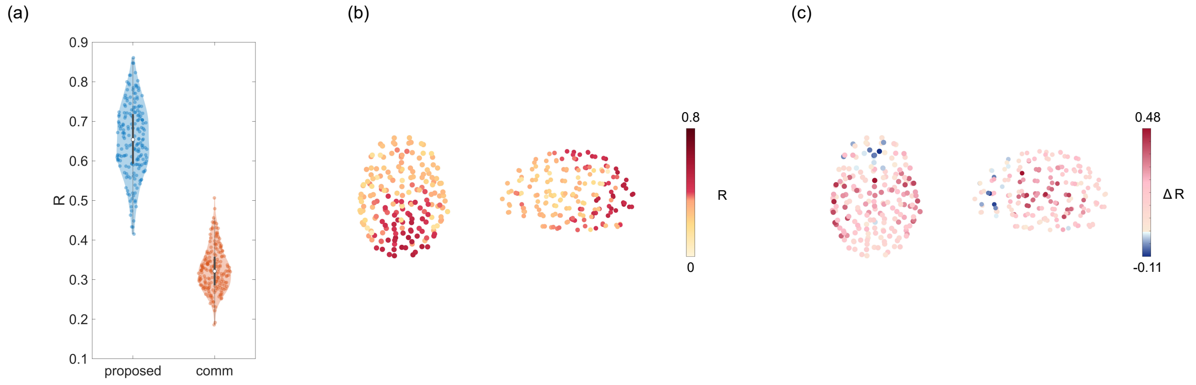

Supplementary Figure 4. Comparison with the communication model in NKI dataset. (**a**) The correlation R of the proposed method vs. the communication model for all individual subjects (n=196 subjects). In each violin plot, the box indicates the interquartile range and the empty circle indicates the median value. Specifically, $R = 0.65 \pm 0.09$ for the proposed approach and $R = 0.33 \pm 0.05$ for the communication model. (**b**) The spatial pattern of SC-FC coupling estimated by the communication model. (**c**) Regional differences in R values of the proposed and communication methods. $70 \pm 10\%$ of brain regions are better explained by the proposed mapping.

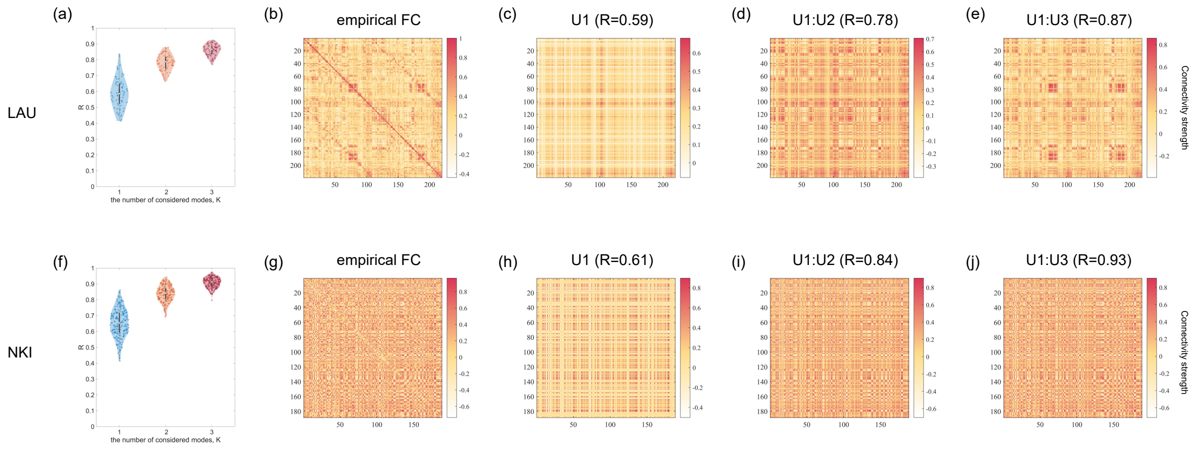

Supplementary Figure 5. Performance of the proposed mapping incorporating different numbers of functional modes. (**a**) Mapping performance for individual subjects in LAU dataset using the proposed method with different number of functional modes (K) under consideration. Higher R values indicate better mapping performance. In each violin plot, points represent individual subjects in LAU dataset (n=69 subjects), the box indicates the interquartile range and the empty circle indicates the median value. Specifically, $R = 0.59 \pm 0.09$ for $K = 1$; $R = 0.78 \pm 0.05$ for $K = 2$; and $R = 0.86 \pm 0.03$ for $K = 3$. (**b**) The empirical FC for a representative subject in LAU dataset. (**c-e**) The estimated FC using our method with different numbers of functional eigenmodes under consideration (from $K = 1$ to $K = 3$). The Pearson R values between the empirical and estimated FC are reported in the sub-captions. (**f**) Mapping performance for individual subjects in NKI dataset (n=196 subjects) using the proposed method with different number of functional modes (K) under consideration. Specifically, $R = 0.65 \pm 0.09$ for for $K = 1$; $R = 0.84 \pm 0.05$ for $K = 2$; $R = 0.91 \pm 0.03$ for $K = 3$. (**g**) The empirical FC for a representative subject in NKI dataset. (**h-j**) The estimated FC using our method with different numbers of functional eigenmodes under consideration (from $K = 1$ to $K = 3$). The Pearson R values for estimated FC are reported in the sub-captions.

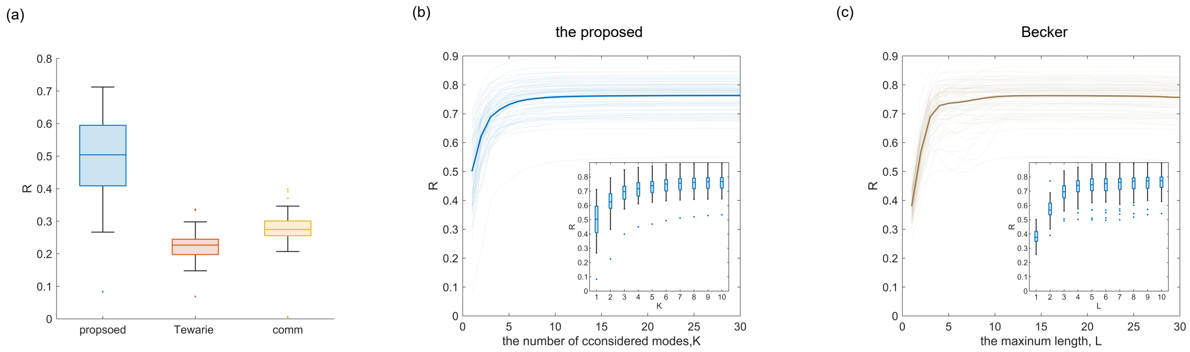

Supplementary Figure 6. Comparison of SC-FC mappings for HCP dataset. We exploit the HCP dataset (n=78 subjects) that contains two sessions of fMRI data to evaluate out-of-sample performance. Specifically, we learn the parameters of subject-specific mapping using the fMRI data from the first session and evaluate the performance on the second session. The mapping performance is quantified by Pearson R between the estimated and empirical FC matrices. In each boxplot, the box indicates the interquartile range (IQR), the horizontal line indicates the median value, and the whiskers cover the upper and lower bounds of $1.5 \times IQR$ (25th and 75th percentiles). Outliers are beyond the whiskers, indicated by dots. (**a**) illustrates the results for the proposed method (with the first functional mode under consideration), Tewarie et al., and the communication model. (**b**) illustrates the evolution of correlation R for the proposed method when we vary the number of considered functional modes (denoted by the parameter K). (**c**) illustrates the evolution of correlation R for Becker et al. when we vary the maximum length of the walks (denoted by the parameter L).

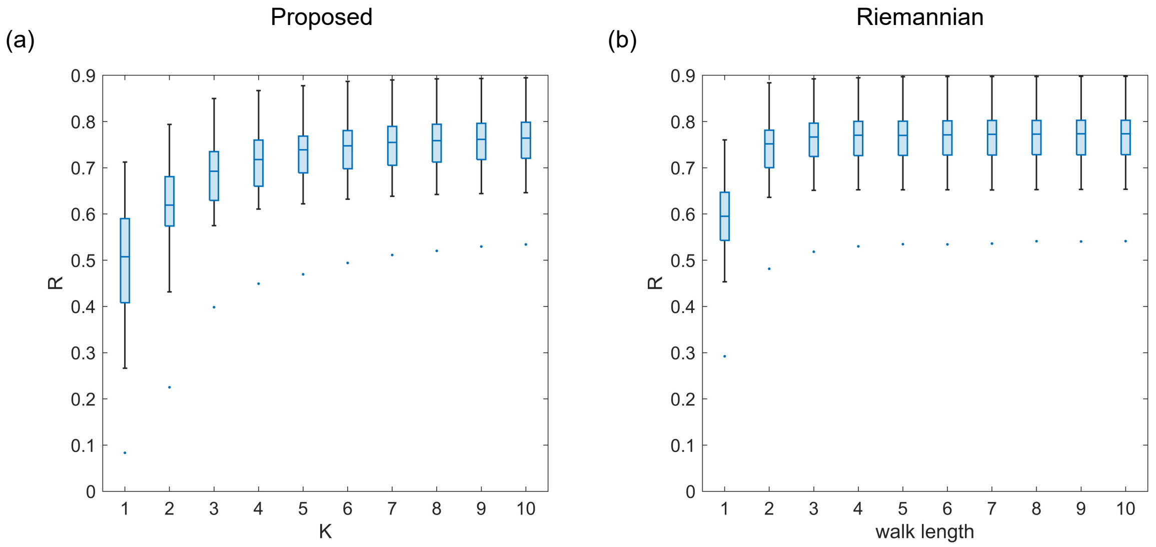

Supplementary Figure 7. Comparison with the Riemannian approach in HCP dataset. (**a**) The mapping performance for individual subjects (n=70 subjects) using our proposed approach with different numbers of functional eigenmodes under consideration (from $K = 1$ to $K = 10$). Metrics of performance are Pearson R between the estimated and empirical FC matrices, excluding the diagonal entries. In each boxplot, the box indicates the interquartile range (IQR), the horizontal line indicates the median value, and the whiskers cover the upper and lower bounds of $1.5 \times IQR$ (25th and 75th percentiles). Outliers are beyond the whiskers, indicated by dots. (**b**) The mapping performance for individual subjects (n=70 subjects) using the Riemannian approach with the maximum walk length varying from 1 to 10.

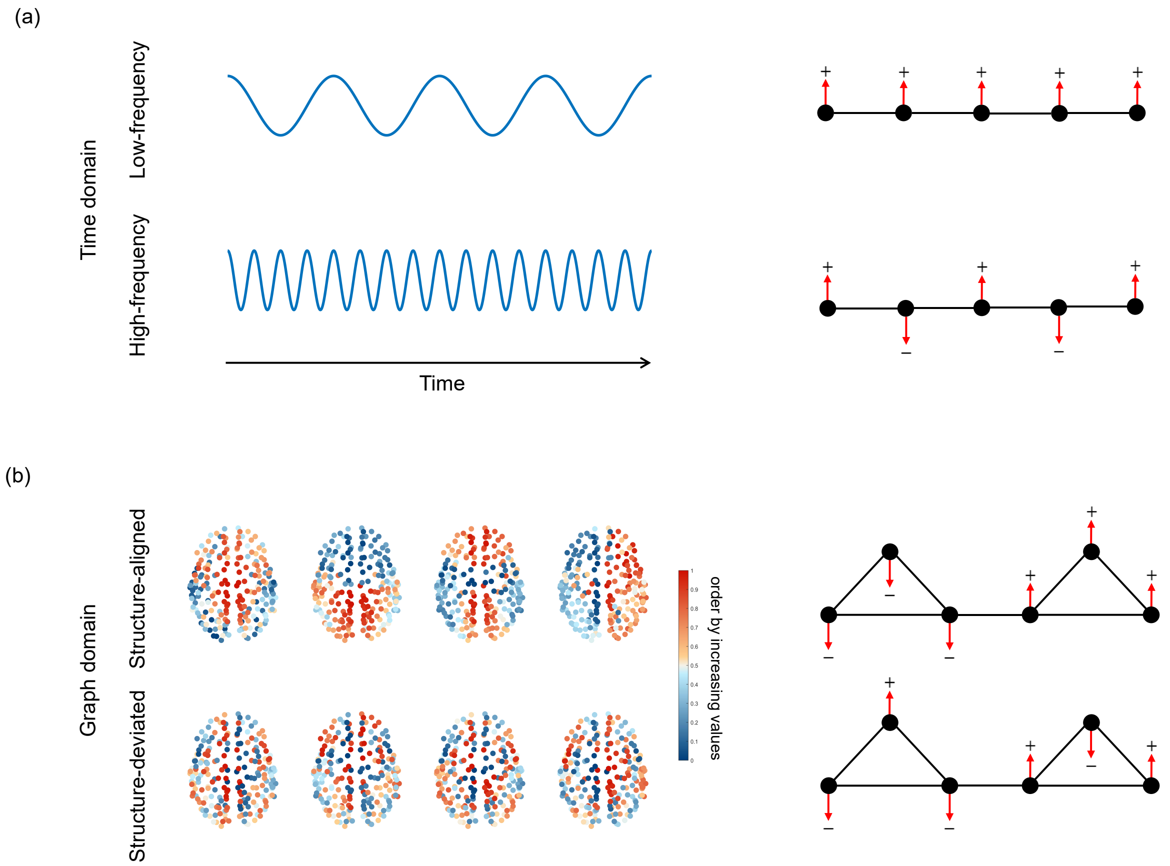

Supplementary Figure 8. The frequency of temporal signals versus the alignment of structural eigenmodes (**a**) In the time domain, low-frequency signals vary slowly along the time dimension, whereby data points that are close in time have similar values; in contrast, high-frequency signals change fast over time, whereby data points may have very dissimilar values even if they are at adjacent time moment. In this way, the frequency of temporal signals reflects to what extent they are time-dependent. (**b**) Analogously, low-frequency/structure-aligned eigenmodes (i.e., those with positive structural eigenvalues) vary smoothly across the graph (here, the structural connectome), whereby nodes that are tightly connected tend to have similar values; in contrast, high-frequency/structure-deviated eigenmodes (i.e., those with negative

structural eigenvalues) exhibit fine-grained variations across the graph, whereby nodes may have very different values even if they are adjacent in the graph. Thus, just as the temporal frequency of signals reflects their dependence on time, the alignment of structural eigenmodes reflects the degree to which they are constrained by the underlying anatomical connectome.

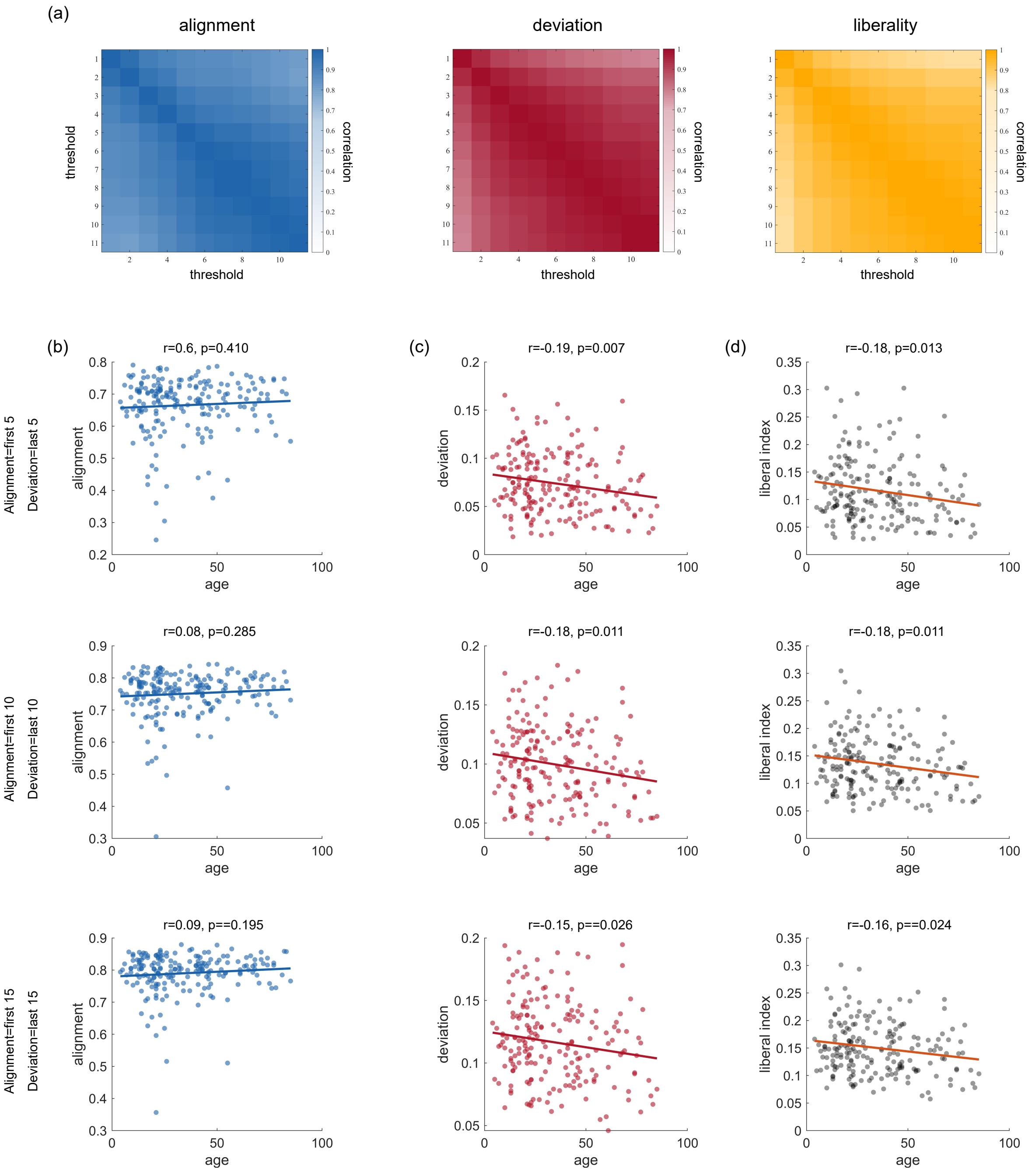

Supplementary Figure 9. Sensitivity analysis of threshold selection. The results in the main text are reported with the threshold K_L and K_H equal to 10, that is, we use the first 10 struc-

tural eigenmodes to represent anatomy-aligned functional components and the last 10 structural eigenmodes to represent anatomy-deviated functional components. To test the robustness of the results, we repeat analyses under values of K_L and K_H from five below to five above the default values. (**a**) The correlation matrices among functional alignment, functional deviation, and structure-function liberality (right panel) across different thresholds. We find these measures exhibit high stability, with the correlation coefficient $r = 0.92 \pm 0.06$ for functional alignment, $r = 0.93 \pm 0.06$ for functional deviation, and $r = 0.94 \pm 0.05$ for structure-function liberality. (**b**) Age-related variations in functional alignment across different thresholds. (**c**) Age-related variations in functional deviation across different thresholds. (**d**) Age-related variations in structure-function liberality across different thresholds. One can notice that the results are robust to different choices of thresholds.
