# Supplementary material for: Eigenmode-based approach reveals a decline in brain structure–function liberality across the human lifespan
Source: Commun Biol. 2023 Nov 7;6:1128. doi: 10.1038/s42003-023-05497-4 (PMC10630517; doi:10.1038/s42003-023-05497-4)
Supplement: Supplementary file 4 — Reporting Summary [file 42003_2023_5497_MOESM4_ESM.pdf]

nature portfolio

Corresponding author(s): Shaoting Tang, Xin Wang

Last updated by author(s): Oct 19, 2023

Reporting Summary

Nature Portfolio wishes to improve the reproducibility of the work that we publish. This form provides structure for consistency and transparency in reporting. For further information on Nature Portfolio policies, see our Editorial Policies and the Editorial Policy Checklist.

Statistics

For all statistical analyses, confirm that the following items are present in the figure legend, table legend, main text, or Methods section.

n/a	Confirmed	
☐	☒	The exact sample size (n) for each experimental group/condition, given as a discrete number and unit of measurement
☐	☒	A statement on whether measurements were taken from distinct samples or whether the same sample was measured repeatedly
☐	☒	The statistical test(s) used AND whether they are one- or two-sided<br>*Only common tests should be described solely by name; describe more complex techniques in the Methods section.*
☒	☐	A description of all covariates tested
☐	☒	A description of any assumptions or corrections, such as tests of normality and adjustment for multiple comparisons
☐	☒	A full description of the statistical parameters including central tendency (e.g. means) or other basic estimates (e.g. regression coefficient) AND variation (e.g. standard deviation) or associated estimates of uncertainty (e.g. confidence intervals)
☐	☒	For null hypothesis testing, the test statistic (e.g. F, t, r) with confidence intervals, effect sizes, degrees of freedom and P value noted<br>*Give P values as exact values whenever suitable.*
☒	☐	For Bayesian analysis, information on the choice of priors and Markov chain Monte Carlo settings
☒	☐	For hierarchical and complex designs, identification of the appropriate level for tests and full reporting of outcomes
☐	☒	Estimates of effect sizes (e.g. Cohen's d, Pearson's r), indicating how they were calculated

Our web collection on statistics for biologists contains articles on many of the points above.

Software and code

Policy information about availability of computer code

Data collection	No software was used for data collection.
Data analysis	The structural connectomes for HCP are constructed through the MRtrix3 software [\url{http://www.mrtrix.org/}]. The code for the spin test is publicly available at \url{https://github.com/spin-test/spin-test}.

For manuscripts utilizing custom algorithms or software that are central to the research but not yet described in published literature, software must be made available to editors and reviewers. We strongly encourage code deposition in a community repository (e.g. GitHub). See the Nature Portfolio guidelines for submitting code & software for further information.

Data

Policy information about availability of data

All manuscripts must include a data availability statement. This statement should provide the following information, where applicable:

- Accession codes, unique identifiers, or web links for publicly available datasets
- A description of any restrictions on data availability
- For clinical datasets or third party data, please ensure that the statement adheres to our policy

The datasets supporting the current study are publicly available. The Lausanne (LAU) dataset can be accessed at https://zenodo.org/record/2872624#.YTR9lI4zaUl. The Nathan Kline Institute (NKI)/Rockland Sample public dataset can be accessed at: http://fcon_1000.projects.nitrc.org/indi/pro/nki.html. The Human Connectome Project (HCP) data can be accessed at: https://www.humanconnectome.org/study/hcp-young-adult.

Human research participants

Policy information about studies involving human research participants and Sex and Gender in Research.

Reporting on sex and gender	LAU: M/F 43/27.<br>NKI: M/F 114/82<br>HCP: M/F 32/46.<br>Since the authors of the present study were not involved in the recruitment of subjects, we have no idea whether sex and/or gender of participants was determined based on self-report or assigned (and methodology used). No sex- and gender-based analyses have been performed because the sample size is low and we did not obtain information on individuals' sex or gender.
Population characteristics	or LAU, the dataset was collected from a cohort of 70 participants (28.8±9.1 years old). For NKI the dataset was collected from a cohort of 196 participants (35±20 years old). For HCP, the dataset consisted of 78 participants (22-36 years old).
Recruitment	The authors of the present study were not involved in the recruitment. Therefore, the authors are not knowledgeable about possible self-section biases or other biases associated with the data collection of the LAU, NKI, and HCP cohorts. Given the consistency of the results across the three datasets, we would estimate that potential biases related to recruitment would not likely impact the main results of the study.
Ethics oversight	LAU was approved by the Ethics Committee of Clinical Research of the Faculty of Biology and Medicine, University of Lausanne. NKI was approved by the Institutional Review Board. HCP was approved by the Washington University institutional review board.

Note that full information on the approval of the study protocol must also be provided in the manuscript.

Field-specific reporting

Please select the one below that is the best fit for your research. If you are not sure, read the appropriate sections before making your selection.

☒ Life sciences ☐ Behavioural & social sciences ☐ Ecological, evolutionary & environmental sciences

For a reference copy of the document with all sections, see nature.com/documents/nr-reporting-summary-flat.pdf

Life sciences study design

All studies must disclose on these points even when the disclosure is negative.

Sample size	Three independent datasets were used in this study. The first dataset was collected by Department of Radiology, University Hospital Center and University of Lausanne (LAU). This dataset contains 70 participants (note that one subject was excluded due to missing fMRI data and therefore 69 subjects were retained for the individual structure-function mappings). The second one is from the NKI which contains 196 participants. The aim is to test for age-related alterations across the human lifespan. The third one is from the HCP which contains 78 participants. No statistical methods were used to predetermine sample sizes. The replicability of our main results across the two independently collected datasets as well as across individual subjects demonstrate the stability of our findings.
Data exclusions	For LAU, one subject was excluded due to missing fMRI data. For NKI, no data were excluded from the analysis. For HCP, we removed individuals with high motion (mean framewise displacement > 0.25mm or max framewise displacement > 2mm), and finally retained 78 subjects for the subsequent analysis.
Replication	The results were replicable across three independently collected datasets (LAU, NKI, HCP) and different thresholds of structure-aligned and structure-deviated portions.
Randomization	Subjects were not partitioned into groups. Data from each dataset (LAU, NKI, HCP) were analyzed separately so as to not mix data across acquisition machines and acquisition parameters.
Blinding	No blinding was applied to this study. As the study only used resting-state data and there was no experimental manipulation, blinding was not relevant to the analyses reported in this study .

Reporting for specific materials, systems and methods

We require information from authors about some types of materials, experimental systems and methods used in many studies. Here, indicate whether each material, system or method listed is relevant to your study. If you are not sure if a list item applies to your research, read the appropriate section before selecting a response.

Materials & experimental systems

n/a	Involved in the study
☒	☐ Antibodies
☒	☐ Eukaryotic cell lines
☒	☐ Palaeontology and archaeology
☒	☐ Animals and other organisms
☒	☐ Clinical data
☒	☐ Dual use research of concern

Methods

n/a	Involved in the study
☒	☐ ChIP-seq
☒	☐ Flow cytometry
☐	☒ MRI-based neuroimaging

Magnetic resonance imaging

Experimental design

Design type: Resting state

Design specifications: For LAU, 280 functional images, 128 diffusion-weighted volumes and a single b0 volume acquired per subject. For NKI, 10-minute resting state fMRI scan (R-fMRI), 64-direction diffusion tensor imaging scan (2mm isotropic). For HCP, rfMRI data were acquired in four runs of approximately 15 minutes each, two runs in one session and two in another session. Diffusion weighting consisted of 3 shells of b=1000, 2000, and 3000 s/mm2 interspersed with an approximately equal number of acquisitions on each shell.

Behavioral performance measures: No behavioral performance measures

Acquisition

Imaging type(s): functional, structural, diffusion

Field strength: 3T

Sequence & imaging parameters: For LAU, Diffusion spectrum images (DSI) were acquired on a 3-Tesla MRI scanner (Trio, Siemens Medical, Germany) using a 32-channel head-coil. The protocol was comprised of (1) a magnetization-prepared rapid acquisition gradient echo (MPRAGE) sequence sensitive to white/gray matter contrast (1-mm in-plane resolution, 1.2-mm slice thickness), (2) a DSI sequence (128 diffusion-weighted volumes and a single b0 volume, maximum b-value 8,000 s/mm2, 2.2×2.2×3.0 mm voxel size), and (3) a gradient echo EPI sequence sensitive to blood oxygen level-dependent (BOLD) contrast (3.3-mm in-plane resolution and slice thickness with a 0.3-mm gap, TR 1,920 ms, resulting in 280 images per participant).
For NKI, the scan was performed in a Siemens Trio 3T scanner. The protocol consisted of: (1) 10-minute resting state 584 fMRI scan (R-fMRI), (2) 6-direction diffusion tensor imaging (DTI) scan, (3) 64-direction diffusion tensor imaging scan (2mm isotropic), (4) MPRAGE anatomical scan, (5) MPRAGE anatomical scan SHORTER sequence, (6)T2 weighted sequence.
For HCP, Structural MRI: 3D MPRAGE T1-weighted, TR=2400 ms, TE=2.14 ms, TI=1000ms, flip angle=8°, FOV=224× 224, voxel size=0.7mm isotropic. Diffusion-weighted MRI: spin-echo EPI, TR=5520ms, TE=89.5ms, flip angle=78°, FOV =210×180, 3 shells of b=1000, 2000, 3000smm−2 with 90 directions plus 6 b=0 acquisitions. Four runs of 15min resting-state fMRI: gradient-echo EPI, TR=720 ms, TE=33.1ms, flip angle=52°, FOV=208×180, voxel size=2mm isotropic.

Area of acquisition: Whole-brain

Diffusion MRI: ☒ Used ☐ Not used

Parameters: For LAU, 128 diffusion-weighted volumes and a single b0 volume, maximum b-value 8,000 s/mm2, 2.2x2.2x3.0 mm voxel size. For NKI, 64-direction diffusion tensor imaging scan (2mm isotropic) with b=1000 s/mm2. For HCP, 3 shells of b=1000, 2000, 3000 s/mm2 with 90 directions plus 6 b=0 acquisitions.

Preprocessing

Preprocessing software: For LAU, initial signal processing of all MPRAGE, DSI, and rs-fMRI data was performed using the Connectome Mapper pipeline (Daducci et al., 2012). Gray and white matter were segmented from the MPRAGE volume using freesurfer (Desikan et al., 2006). For NKI, FSL and Diffusion Toolkit. For HCP, Python and MRtrix3.

Normalization: For LAU, the diffusion acquisition was used as the reference space and the tissue masks have been registered to the b0 volume using appropriate registration methods (Daducci et al., 2012). For HCP, data have been aligned across modalities and across subjects to Montreal Neurological Institute (MNI) standard space using appropriate volume-based and surface-based registration methods (see HCP documentation for details). For NKI, register with FSL FLIRT to MNI152 average brain.

Normalization template: For LAU, data was analyzed after registering the T1-weighted image to the b0 volume, i.e. the volume acquired in absence of diffusion sensitising gradients. For HCP, data was analyzed after alignment to the MNI template. For NKI, MNI152 average brain.

Noise and artifact removal

For LAU, fMRI volumes were corrected for physiological variables, including regression of white matter, cerebrospinal fluid, as well as motion (three translations and three rotations, estimated by rigid body co-registration).
For HCP, fMRI data were denoised using ICA-FIX and Butterworth filter.
For NKI, regress CSF, WM, whole brain signal, six motion parameters, and all temporal derivatives from BOLD data, motion scrubbing of TRs with relative motion displacement greater than 0.5mm or relative BOLD signal intensity change greater than 0.5% (Power et al., 2012)

Volume censoring

censoring only for NKI fmri data

Statistical modeling & inference

Model type and settings

General linear model performed for nuisance regression. Voxelwise timecourses are considered for functional connectivity analysis. NO effect tested.

Effect(s) tested

None, non relevant.

Specify type of analysis: ☐ Whole brain ☒ ROI-based ☐ Both

Anatomical location(s)

For LAU, gray matter was parcellated into 68 cortical nodes according to the Desikan–Killiany atlas (Desikan et al., 2006). These regions of interest were then further divided into 219 approximately equally sized nodes (Cammoun et al., 2012).
For NKI, Craddock 200 atlas (R. C. Craddock et al., 2012) was used to parcellate the cortex into N=188 regions of interest.
For HCP, Schaefer400 atlas (A. Schaefer et al., 2018) was used to parcellate the cortex into N=400 regions of interest.

Statistic type for inference
(See Eklund et al. 2016)

None, non relevant.

Correction

None, non relevant.

Models & analysis

n/a	Involved in the study
☐	☒ Functional and/or effective connectivity
☐	☒ Graph analysis
☐	☒ Multivariate modeling or predictive analysis

Functional and/or effective connectivity

Pearson's correlation

Graph analysis

We built weighted structural connectivity graphs and estimated the connectivity as the density of fibers connecting two regions. A group-level connectome was obtained by averaging all subjects' structural matrices.

Multivariate modeling and predictive analysis

We utilized multilinear regression model to perform regional structure-function prediction. The predictors are Laplacian eigenmodes of the structural connectome. The performance of structure-function prediction is evaluated as the Pearson correlation between the upper triangular entries (excluding the diagonal entries) of the predicted and empirical FC matrices.
